# Revealing the impact of *Pseudomonas aeruginosa* quorum sensing molecule 2’-aminoacetophenone on the human bronchial-airway epithelium and pulmonary endothelium using a human airway-on-a-chip

**DOI:** 10.3389/fimmu.2025.1592597

**Published:** 2025-07-15

**Authors:** Shifu Aggarwal, Arijit Chakraborty, Vijay K. Singh, Stephen Lory, Katia Karalis, Laurence G. Rahme

**Affiliations:** ^1^ Department of Surgery, Massachusetts General Hospital, and Harvard Medical School, Boston, MA, United States; ^2^ Department of Microbiology, Harvard Medical School, Boston, MA, United States; ^3^ Department of Surgery, Shriners Hospitals for Children, Boston, MA, United States

**Keywords:** *Pseudomonas aeruginosa*, 2-aminoacetophenone, mvfR, pqsR, MvfR/pqsABCDE system, airway-on-a-chip, cholesterol biosynthesis

## Abstract

**Background:**

*Pseudomonas aeruginosa (PA)* causes severe respiratory infections utilizing multiple virulence functions. Previous findings on the PA secreted quorum sensing (QS)-regulated small molecule, 2’-aminoacetophenone (2-AA), revealed its impact on immune and metabolic functions, favouring a long-term presence of PA in the host. However, the 2-AA’s specific effects on bronchial-airway epithelium and pulmonary endothelium remain elusive. To evaluate the spatiotemporal changes in 2AA within the human airway, considering endothelial cells as the primary point of contact when the route of lung infection is hematogenic, we utilized the airway-on-achip platform. This dynamic culture system recapitulates critical elements of the human airway microphysiological environment.

**Methods:**

We utilized the microfluidic airway-on-chip platform, lined by polarized primary human pulmonary microvascular endothelial cells (HPMEC) and adjacent primary normal human bronchial epithelial cells (NHBE) obtained from healthy female donors. Cells exposed to 2-AA (20 μm) through continuous flow for 12 hours were used for whole-genome RNA sequencing and analyzed for their responses and potential cross-talk. Transcriptome findings were validated through *in vivo* studies in mice and additional cell culture experiments.

**Results:**

Analyses revealed that 2-AA differentially regulates specific signaling and biosynthesis pathways in epithelial cells, including HIF-1 and pyrimidine signaling, glycosaminoglycan and glycosphingolipid biosynthesis. In endothelial cells, fatty acid metabolism, phosphatidylinositol, and estrogen receptor signaling, as well as proinflammatory signaling pathways, were identified. Significant overlap was found in both cell types in response to 2-AA in genes implicated in immune response and cellular functions. In contrast, we found that genes related to barrier permeability, cholesterol metabolism, and oxidative phosphorylation were differentially regulated in response to 2-AA exposure in the studied cell types. Murine *in vivo* and additional *in vitro* cell culture studies confirmed the accumulation of cholesterol in epithelial cells. Results also revealed that specific biomarkers associated with cystic fibrosis and idiopathic pulmonary fibrosis were modulated by 2-AA in both cell types, with the expression of cystic fibrosis transmembrane regulator being affected only in endothelial cells.

## Introduction

Pathogens have evolved diverse mechanisms to manipulate host cell functions, facilitating their survival and evasion of the host’s immune response. Bacterial quorum sensing (QS), a cell density-dependent conserved system, via the synthesis and secretion of signaling molecules, coordinates various virulence activities in Gram-negative and Gram-positive bacteria and plays an important role in the modulation of immune and metabolic functions (1–6).

*Pseudomonas aeruginosa (PA)*, a recalcitrant ESKAPE pathogen, is notorious for its rapid development of antibiotic resistance and ability to cause acute and chronic infections (7–10). This opportunistic pathogen can infect diverse tissues, including the lungs, as a dominant respiratory pathogen. It employs multiple mechanisms to target and damage epithelial and endothelial cells, including adherence and invasion, secretion of various exotoxins, and inhibition of angiogenesis (11, 12). *PA* can adhere to and invade these cells, surviving intracellularly for extended periods, which leads to progressive damage of endothelial and epithelial tissue (11–13). This pathogen causes acute pneumonia and chronic infections in individuals with compromised lung defenses, such as people with cystic fibrosis, those with bronchiectasis, chronic obstructive pulmonary diseases, and critically ill patients on mechanical ventilation or otherwise immunocompromised patients (14–17). A commonality among these conditions is the impairment of the lung’s innate immunity, characterized by dysfunction of the mucociliary escalator, mucus accumulation, damage to the lung epithelial barrier, and persistent inflammation. These factors collectively heighten susceptibility to infections, disrupt local immune response, and hinder effective treatment (12).

*PA* QS systems, LasR/I, RhlR/I, and MvfR/PqsABCDE, are responsible for the synthesis of various low-molecular-weight signaling molecules that regulate virulence (18). Few of these small molecules have been shown to modulate the host immune response (2, 3, 19–21). Specifically, the MvfR/PqsABCDE system, also called Pqs signaling, regulates the synthesis of the 4-hydroxy-2-alkylquinolines (HAQs), and the non-HAQ molecule, 2’-aminoacetophenone (2-AA) (22–24). 2-AA promotes the formation of *PA* antibiotic-tolerant/persister cells and *lasR* mutations (24–26) and is synthesized and secreted in *P. aeruginosa*-infected human tissues, making it a promising breath biomarker in patients with *PA* infection (27). Small molecules secreted by bacteria at the infection site can enter the bloodstream (28, 29). Once in circulation, these molecules may rapidly reach the pulmonary capillaries, where their first point of contact is the endothelial cells lining the capillary walls (30). Endothelial cells can facilitate translocation and/or elicit a direct response to these bacterial molecules, thereby influencing epithelial cell responses. However, the cascade of events leading to epithelial infection has not been fully characterized. Independently of the dissemination of the bacterial-produced small molecules, infections disseminating via the hematogenous route are common in immunocompromised patients (31).

Our previous studies in *PA* uncovered unique immunometabolic mechanisms by which 2-AA mediates mutual pathogen-host adaptation (2, 19, 32–34). We identified how 2-AA induces host epigenomic reprogramming through metabolic derangement, rewiring immune and metabolic functions to enable tolerance to persistent infection while rescuing the mortality of infected animals (2, 3, 19–21). 2-AA-induced immune tolerization in macrophages leads to metabolic perturbations in mitochondrial functions (3). It impacts the autophagic machinery and lipid biosynthesis to sustain *the* presence of PA in macrophages (3). In skeletal muscle, 2-AA also compromises mitochondrial functions (1, 34), leading to increased oxidative stress and subsequent apoptosis (32). Despite this small molecule’s multifaceted role, its effect on pulmonary function and physiology remains poorly understood.

Microphysiological system leveraging technologies for organ-on-chip engineering have emerged in the last few years (35–37). Studies have shown that with these micro-engineered devices, critical elements of human organ physiology, cell-specific changes associated with disease states, and mechanisms driving therapeutic responses to clinically relevant drug exposures can be recapitulated with high fidelity (38–42). The commercially available Lung-Chip, an airway-on-a-chip platform, provides an engineered “microtissue” that emulates the cell-cell interface, allowing for the controlled application of mechanical forces such as stretch and shear stress, flow, and exposure to *in vivo-relevant* biochemical cues and signals (43–45) as well as readouts with the experimentally desired spatiotemporal resolution. It enables detailed molecular, biochemical, and metabolic studies on lung cells maintained in an epithelial/endothelial interface supported by a human tissue-relevant extracellular matrix, allowing for an *in vivo-relevant* barrier formation. This interface is crucial in preventing the non-specific migration of cells through the membrane pores, enabling the functional maturation of cells within the chip, and closely replicating the transfer of vascular molecules to the epithelium. Therefore, unlike the transwell system, which is a static system, the airway-on-a-chip platform provides a dynamic culture system that emulates *in vivo*-relevant physiological functions. Given the increasing concern over bacterial infections and their effects on pulmonary health, understanding the mechanisms underlying epithelial-endothelial cell interactions during microbial interactions with the host, as well as the impact of secreted bacterial products, is vital for developing new therapeutics and identifying disease biomarkers.

In this study, using the cutting-edge human airway-on-a-chip platform, we investigated the impact of 2-AA on human primary pulmonary microvascular endothelial cells (HPMEC) and normal human bronchial epithelial (NHBE) cells from healthy donors, with a primary focus on pulmonary endothelial cells as the primary point of contact. This may occur in *PA-*disseminated infections when the route of lung infection is through the bloodstream. *PA* actions on endothelial cells compromise vascular integrity and play a significant role in the pathogenesis of infections caused by this versatile pathogen, which potentially contributes to bacteremia in compromised patients (46). Our data revealed multiple common and cell-specific 2-AA-mediated responses that affect various signaling pathways and genes, providing novel insights into the effects of this molecule and informing the development of new therapeutics to mitigate the impact of this pathogen.

## Materials and methods

### Activation of the airway-on-a-chip platform

The human airway-on-a-chip platform used in this study is the 2-channel microfluidic Chip-S1 Organ Chip device fabricated by Emulate Inc. It consists of two parallel channels: the mucociliary airway epithelium at the top and a microvascular endothelium at the bottom channel, separated by a human tissue-relevant extracellular matrix. The PDMS membrane was activated one day prior to introducing primary human lung microvascular endothelial cells (HPMEC) and normal human bronchial epithelial cells (NHBE) from healthy female donors using ER1 (Emulate Reagent 1) and ER2 (Emulate Reagent 2) reagents (Emulate, 10465). A total of 50 μL (0.5 mg/mL) ER1 solution, resuspended in ER2, was introduced into the top and bottom channels through the corresponding inlets using a 200 μL micropipette. Chips were incubated under UV light for 10 mins, followed by a 3 min incubation at room temperature and another 10 min UV treatment. Subsequently, both channels were washed with ER2, then with cold 1X DPBS (Dulbecco’s Phosphate Buffered Saline) and coated overnight at 37°C with 50 μL solution of 50 μg/mL Fibronectin (Corning, 354008), 50 μg/mL Laminin (Millipore Signa, 05-23-3703 1MG) and 100 μg/mL Collagen I (Advanced BioMatrix (50-360-233) added in the inlets of the top and bottom channel.

### Establishment of the HPMEC and NHBE cell cultures in the airway-on-a-chip and treatment with 2-AA

Following the overnight coating, chips were washed twice with 1X PBS. The endothelial cells (HPMEC) (Millipore Sigma, C-12281), at a concentration of 6 X 10^6^ cells/mL (∼30 μL) diluted in Endothelial Cell Growth Medium MV 2 (Promocell, C-22221 and Growth Medium MV 2 Supplement Pack C39221), were introduced through the inlets of the bottom channel. Chips were inverted, and cells were left to adhere for 2 hrs at 37°C and 5% CO_2_. Chips were then inverted back and epithelial (NHBE) cells (Lonza, CC-2570, 0000615564) at a concentration of 3 X 10^6^ cells/mL (approximately 40 μL), diluted in epithelial growth medium (PromoCell, C-21260, and Supplement Pack Airway Epithelial Cell GM C39160), were introduced into the inlet of the top channel.

To remove the non-adherent cells the following day, the upper channel was gently washed with epithelial growth medium and the bottom channel with endothelial cell growth medium MV 2. The chips were attached to Pod™ Portable Modules (Emulate Inc., 10153) and connected to the automated cell culture system Zoë™ at a flow rate of 30 μL/h in both channels to establish a monolayer for 7 days. Culture media was refreshed every two days. From day 8 through day 14, the epithelial channel was switched to Air-Liquid Interface (ALI) by removing the media from the top channel, allowing cell epithelial cell polarization. The bottom channel was continuously perfused with a fresh medium mimicking human vasculature using the PneumaCult™-ALI Medium (Stemcell, 05002), supplemented with 10 ng/mL VEGF and 1 µg/mL Vitamin C, and the flow rate was set at 40 µL/hr. At day 14, 20 μM of the 2-AA compound (Sigma, A37804) was added to the ALI medium (bottom channel) with continuous flow for 20 hrs. After treatment, the medium was removed from the bottom channel, and chips were used to extract or stain RNA.

### RNA isolation from the chips

The top and bottom channels of the chip were lysed, and NHBE and HPMEC cells were collected separately. Both channels were rinsed once with 200 μL of ice-cold phosphate-buffered saline (PBS). Subsequently, we blocked the inlet and outlet of the channel opposite the one containing the cells of interest using empty 200 μL tips. We gently rewashed the channel of interest with 200 μL of ice-cold phosphate-buffered saline (PBS). Once the washing was completed, the PBS was gently aspirated from the channel, leaving it dry. The outlet port of the channel of interest was blocked with an empty 200 μL tip. The tip was not pushed entirely against the bottom of the channel, allowing for a smooth flow of lysis buffer in and out of the pipette tip. Then, we introduced 70 μL of lysis buffer (Qiagen, 74004) into the channel of interest (through the inlet port) using a 200 μL tip. The cell lysate was collected in an RNase-free Eppendorf Tube^®^ and placed on ice or stored at -80°C. RNA extraction from the cells was isolated using the RNeasy Micro Kit (Qiagen, 74004). Genomic DNA was removed using the RNase-Free DNase Set (Qiagen, 1023460).

### RNA sequencing and data analysis

RNA isolated from untreated and 2-AA-treated (n = 2) HPMEC and NHBE cells was sequenced using an Illumina platform (MGH core facility). rRNA from the samples was depleted using a ribosomal depletion kit, and sequencing was performed (paired-end reads of 100 bp) on an Illumina HiSeq 2500 platform.

The RNA sequencing data were analyzed using the cloud-computing server of Galaxy (usegalaxy.org), an open-source, web-based platform for next-generation sequencing analysis (47). The paired-end reads obtained were quality-controlled using FastQC (Galaxy version 0.74). Trimming of the 3’ adaptor and the low-quality reads was removed using the Cutadapt tool (Galaxy version 4.8). The trimmed paired-end reads were joined using the concatenate tool (Galaxy version 9.3). The reads were mapped to Homo sapiens (release 38) reference sequence (GRCh38) using STAR alignment (galaxy version 2.7.11a). Genome annotations for the human reference genome were obtained from the UCSC Genome Browser Gateway. Reads mapped to the GRCh38 were counted using featureCounts (Galaxy version 2.0.3) with default parameters.

Differential gene expression analysis was performed using DeSeq2 version 1.40.2 with default parameters. Genes with an increase or decrease of at least 0.5 or -0.5 log2 fold change and a maximum p-value of 0.05 were considered as significantly regulated genes. Volcano plots were generated using graphpad prism software. Heatmaps were created using Galaxy Heatmap2 (version 3.1.3.1). The Reactome pathway enrichment analyses of the significantly regulated genes were visualized using the DAVID web server (https://david.ncifcrf.gov).

### Immunostaining

HPMEC cells were seeded at a density of 5 X 10^4^ cells per well on three-well chambered cover glass slides and cultured in an Endothelial Cell Growth Medium MV 2 (Promocell, C-22221 and Growth Medium MV 2 Supplement Pack C39221). Cells were grown for 48–72 hrs until they reached 90-95% confluency. Confluent monolayers were treated with 1X PBS (pH 7.4) or 200 μM 2-AA in PBS for 20 hr at 37°C with 5% CO_2_. Following treatment, cells were fixed with 4% paraformaldehyde in 1X PBS (pH=7.4), washed with 1X PBS (pH=7.4), and incubated with blocking solution (2.5% bovine serum albumin (BSA), (0.05% Triton X-100, and 1X PBS (pH 7.4)) for 1 hr at room temperature. Primary antibody against MMP-9 (Cell Signaling Technology,40543S; 1:100) was added and incubated overnight at 4°C. After washing with PBST buffer, the cells were incubated in fluorescent-labeled secondary antibodies, followed by DAPI (1:10,000) staining at room temperature, and washed in 1X PBS (pH 7.4) three times for 5 min each. Images were acquired using confocal microscopy (Nikon ECLIPSE Ti2; NIS-Elements 5.21; Nikon Instruments Inc., Tokyo, Japan). The fluorescence intensities of MMP-9 (red channel) and DAPI (blue channel) were measured using ImageJ software. The fluorescence intensity in relative expression was calculated by normalizing the MMP-9 with the DAPI stain.

### Leukocyte adhesion assay

HPMECs were seeded at a density of 5 X 10^4^ cells/well onto three-well chambered cover glass slides (Ibidi) and incubated at 37°C with 5% CO_2_. Upon reaching 90-95% confluency, the cells were treated with 2-AA at a final concentration of 200 μM and incubated for 20 hrs at 37°C with 5% CO_2_. After treatment, VCAM-1 (Cell Signaling Technology, 13662T) antibody was added at a dilution of 1:1000 for 1 hr at 37°C. THP-1 cells (2 X 10^4^ cells/well) previously stained with CellTracker green dye (Invitrogen, C2925) were added to the HPMEC monolayer and incubated for 2 hr at 37°C to allow for transmigration. Following incubation, cells were fixed with 4% paraformaldehyde in 1X PBS (pH = 7.4) for 15 mins at room temperature. Slides were washed with 1X PBS (pH 7.4) and incubated with blocking solution (2.5% bovine serum albumin (BSA), 0.05% Triton X-100, and 1X PBS (pH 7.4)) for 1 hr at room temperature. For immunofluorescence staining, cells were incubated overnight at 4°C with a primary anti-VCAM-1 antibody (1:1000 dilution). After washing three times with PBST buffer, cells were incubated in fluorescent-labeled secondary antibodies, followed by DAPI (1:10,000) staining and further washing as above. Confocal images were acquired as above. The fluorescence intensities of VCAM-1 (red channel), THP-1 (green channel), and DAPI (blue channel) were measured using the ImageJ software. The fluorescence intensity in relative expression was calculated by normalizing the VCAM-1 and THP-1 signals with the DAPI stain.

### Multiplex cytokine analysis

HPMECs were seeded at a density of 5 X 10^5^ cells/well in 6-well plates containing endothelial cell growth medium MV 2. After reaching 80-90% confluence, cells were treated with 200 μM of 2-AA or PBS control for 20 hrs at 37°C with 5% CO_2_. Following treatment, culture supernatants were collected and centrifuged at 2,000 rpm for 5 mins at 4°C to remove cellular debris. The cell-free supernatants were processed directly for cytokine profiling. Following the manufacturer’s instructions, inflammatory cytokine levels were quantified using the Bio-Plex Human Essential Immune Response Panel (13-plex) (BioLegend LEGENDplex™, 740929). Data acquisition was performed using BD Accuri C6 Plus flow cytometer, and cytokine concentrations were determined by generating a standard curve with the provided standards through the LegendPlex software.

### Airway-on-a-chip staining

Following media removal from both channels, chips were stained with 1 μM of a working solution of the mitotracker dye (Fisher, M7512) and incubated for 15 mins at 37°C with 5% CO_2_, protected from light. The chips were fixed with 4% paraformaldehyde (PFA) in PBS and kept for 30 mins in the dark at room temperature with 200 μl tips in the ports. Chips were washed once with 1X PBS for 5 mins, followed by a 10-min wash with 1X PBST. The chips were stained with filipin (Sigma, SAE0087; 1:2000 dilution) and nuclear green LCS1 (Abcam, ab138904; 1:4000 dilution) in PBS for 1 hr at room temperature in the dark. Chips were rinsed three times with 1X PBS, and visualization was done by confocal microscopy (Nikon ECLIPSE Ti2; NIS-Elements 5.21; Nikon Instruments Inc., Tokyo, Japan). The fluorescence intensity of the mitotracker (red channel), Filipin (blue channel), and DAPI (green channel) was measured using the ImageJ software. The fluorescence intensity in relative expression was calculated by normalizing the Mitotracker with LCS1 and Filipin to the corresponding LCS1 stain.

### Quantification of cholesterol from the chip effluent

Effluent from the endothelial channel, maintained at the air-liquid interface (ALI), was collected before treatment (0 hr) and after 20 hrs of treatment with either 20 μM 2-AA or PBS. Following the manufacturer’s instructions, the cholesterol levels in the collected effluents were quantified using the Amplex Red Cholesterol Assay Kit (Fisher, A12216). Cholesterol concentrations in the 2-AA-treated and the PBS-treated groups were normalized using the standard curve generated from known cholesterol concentrations.

### Mice treatment and lung tissue stainings

Six-week-old female C57BL/6 mice received a single intraperitoneal (IP) injection of 100 μl of 2-AA in PBS (6.75 mg/kg body weight). The control group received 100 μl of PBS. After 4 days, the mice were euthanized, and the lungs were perfused with normal saline via the trachea. The lungs were inflated with 10% neutral buffered formalin, the trachea was tied off, and the lungs were removed for overnight fixation in formalin. Fixed lungs were dehydrated and embedded in paraffin wax using disposable base molds (Fisher HealthCare, 22363553). Consecutive 5 μm sections were generated from the lung tissue blocks using a Leica RN2025 microtome. Lung sections were mounted onto glass microscopic slides and stored at room temperature until use. Slides were deparaffinized and rehydrated using the 1X antigen retrieval solution. Sections were washed twice with 1X PBS (pH 7.4) and incubated with 10% goat serum for 1 hour at room temperature to block non-specific binding.

To assess cholesterol biosynthesis, we used the anti-HMGCR (Abcam, ab242315) primary antibody (1:200 in 10% goat serum) overnight at 4°C, protected from light. The slides were washed three times with 1X PBS and incubated with a fluorescently labelled secondary antibody (Abcam, ab175700; 1:200 in 10% goat serum) for 1 hr at room temperature. The tissue nuclei were counterstained with DAPI (Sigma, D9542; 1:10,000) for 5 mins at room temperature, followed by three washes with 1X PBS (pH 7.4). Stained tissue sections were examined using a confocal microscope (Nikon ECLIPSE Ti2; Nikon Instruments Inc., Tokyo). Images were captured from at least six random fields per section. Fluorescence intensity was quantified using ImageJ software.

For the haematoxylin-eosin (H&E) staining, the lung tissue sections (5 μm) were stained with H&E, dehydrated through a series of graded alcohols, and cleared in xylene. Finally, the sections were mounted using a DPX (dibutyl phthalate in xylene) mounting medium. The mounted sections were visualized and imaged using an Echo Rebel microscope.

### Filipin staining on chamber slides

NHBE and HPMEC cells were grown to 95% confluency on chamber slides (Ibidi, 80826). Cells were treated with PBS (control) or 200 μM 2-AA for 24 hrs at 37^°C^, 5% CO2. After treatment, cells were washed twice with PBS (pH 7.4) and fixed with 4% paraformaldehyde for 15 mins at room temperature. A working solution was prepared by diluting filipin III (Sigma, SAE0087; 1:2000) in PBS. Cells were stained with the filipin working solution for 1 hr at room temperature in the dark. Stained cells were visualized by confocal microscopy (Nikon ECLIPSE Ti2; NIS-Elements 5.21; Nikon Instruments Inc., Tokyo, Japan). Images were captured from at least six random fields per replicate. The fluorescence intensities of the green and blue channels were measured using ImageJ software. The fluorescence intensity in relative expression was calculated by normalizing the blue fluorescence of Filipin with the corresponding green fluorescence values representing the LCS1 stain.

### Quantification of total cholesterol in HPMEC and A549 cell lines

The HPMEC cells were cultured in a T-75 flask using Endothelial Cell Growth Medium MV 2 (Promocell, C-22221 and Growth Medium MV 2 Supplement Pack C39221), supplemented with 1X penicillin/streptomycin (Gibco). Human lung epithelial A549 cells were maintained in T-75 flasks containing RPMI-1640 medium (Gibco) supplemented with 10% heat-inactivated FBS (endotoxin-free, Certified FBS; Invitrogen) and 1X penicillin/streptomycin (Gibco). Both cell lines were maintained at 37°C with 5% CO2. Cells were used between passages 3 and 4.

HPMEC and A549 cells were seeded in 100 mm Petri dishes with 95% confluency. Cells were treated with 200 μM 2-AA for 24 hrs, with PBS-treated cells serving as the control. The supernatant was collected for extracellular cholesterol measurement at 0 hrs and 24 hrs after treatment. The cells were collected after the treatment to measure the total cell cholesterol. The cells were centrifuged at 2000 rpm for 5 mins, and the supernatant was discarded. The cell pellet was resuspended in ice-cold RIPA lysis buffer (Cell Signalling Technology, USA) containing a protease inhibitor cocktail (Millipore 1183580001) and was kept on ice for 1 hr. The cells were centrifuged at 5000 rpm for 15 mins, and the supernatant was collected to quantify the total cell cholesterol. Extracellular cholesterol levels and total cellular cholesterol were quantified using the Amplex Red Cholesterol Assay Kit (Fisher, A12216) according to the manufacturer’s instructions. Cholesterol concentrations were normalized using a standard curve created with known concentrations of cholesterol.

### MTT assay for cytotoxicity

The cytotoxicity of 2-AA was measured using the 3-(4,5-dimethylthiazol-2-yl)-2,5-diphenyltetrazolium bromide (MTT) assay (Sigma, M5655). HPMECs were seeded at a density of 5 × 10^4^ cells/well in a 96-well plate (Corning) and allowed to adhere overnight. Cells were then treated with various concentrations of 2-AA (20-200 μM) for 20 hrs at 37°C with 5% CO_2_. After treatment, 100 μL of MTT solution (0.5 mg/mL in media) was added to each well and incubated for 2 hrs at 37°C to allow conversion to the insoluble purple formazan. The medium was aspirated, and formazan was solubilized with 100 μL of 95% isopropanol containing 5% formic acid for 15 mins. The absorbance was then measured at 570 nm using a Tecan plate reader. Cell viability was calculated as a percentage relative to cells treated with control, phosphate-buffered saline (PBS).

### Statistical analysis

All statistical analyses were performed using GraphPad Prism 9 software except for RNA-sequencing analysis. Wherever applicable, at least four or five independent experiments were performed. Data was analyzed using a two-tailed t-test and ordinary one-way analysis of variance (ANOVA) with Tukey’s *post hoc* test. For all experiments, p-values of less than 0.05 were considered statistically significant.

## Results

### 2-AA affects the gene expression of human pulmonary microvascular endothelial cells and bronchial airway epithelial cells

**Figure 1:**
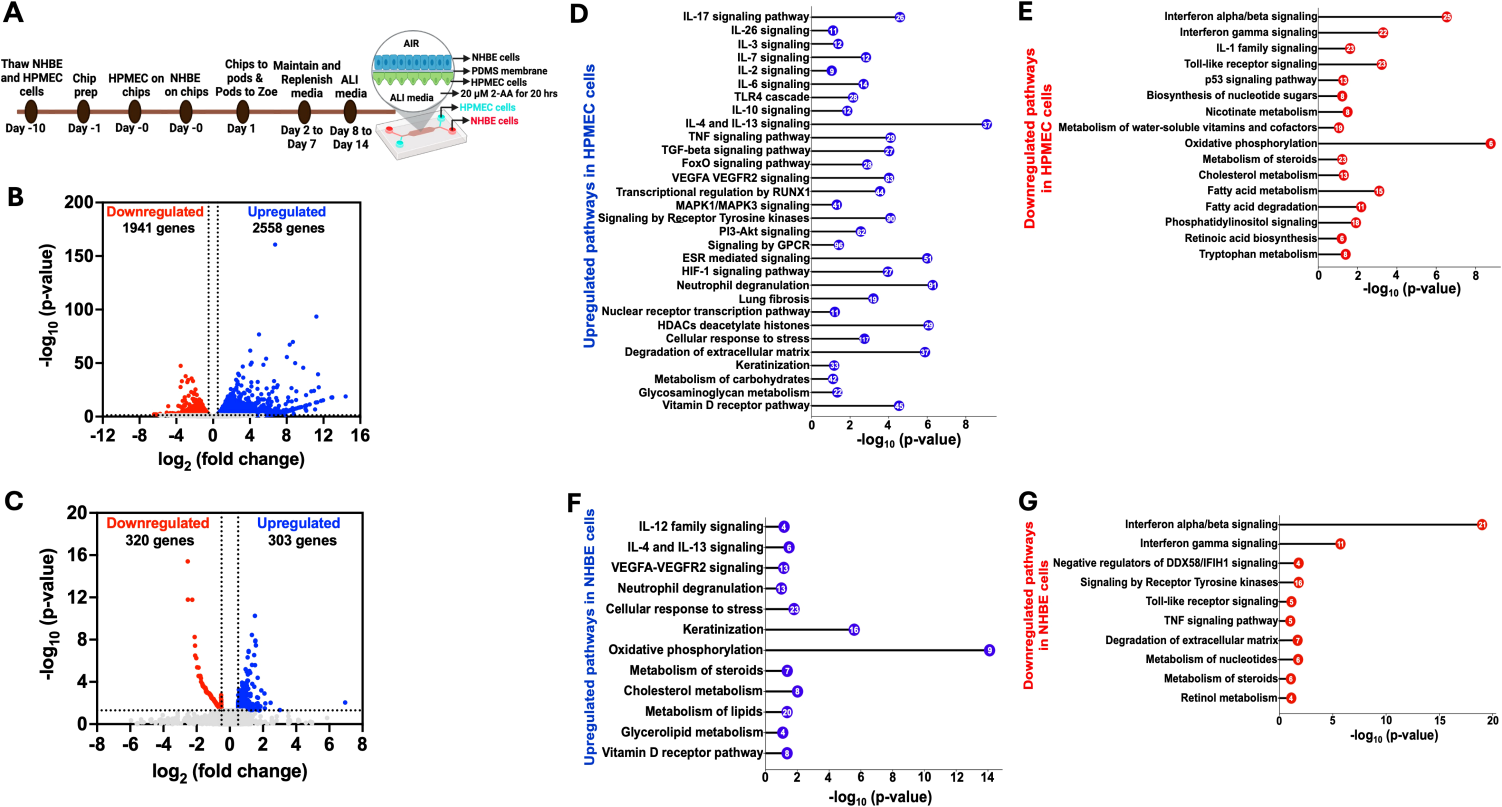
Airway-on-a-chip RNA-sequencing analysis of 2-AA-treated endothelial and epithelial cells reveals multiple pathways to be differentially regulated by this quorum sensing molecule **(A)** Overview of the timeline of preparation and treatment of 2-AA of human pulmonary microvascular endothelial (HPMEC) and normal bronchial epithelial (NHBE) cells in a lung-on-chip setting. Volcano plot of the differentially expressed genes (DEGs) in HPMEC **(B)** and NHBE **(C)** cells after 20 hrs of continuous flow of 20 µM 2-AA. The blue dots represent genes that are upregulated (log_2_fold change>0.5), red dots for downregulated (log_2_fold change <-0.5), and grey dots for genes that are not significant. The two vertical dashed lines indicate log2-fold change > 0.5 and log2-fold change < -0.5. The horizontal dashed line indicates the significance of -log_10_ (p-value) =1.3. **(D-G)** Horizontal lollipop plots showing Reactome pathway enrichment analysis of upregulated and downregulated pathways from the differentially regulated DEGs in human HPMEC and NHBE cells and compared to untreated cells with -log_10_(p-value) =1.3 on the x-axis. The numbers in the circles represent the count of genes for each pathway. DESeq2 determined the log2 fold change values used in volcano plots. The -log_10_ (p-value) of 1.3 corresponds to a p-value of less than 0.05, as determined by Deseq2. The Reactome pathway enrichment analysis was done using the DAVID web server. The RNA sequencing analysis was performed in two independent experiments (n = 2).

Using the human airway-on-a-chip platform, we assessed the effect of 2-AA on human pulmonary microvascular endothelial cells (HPMEC) and normal human bronchial airway epithelial cells (NHBE). **Figure 1A** illustrates the platform, timeline, and steps of preparation and 2-AA treatment of primary human cells, with NHBE cells in the top channel and HPMEC cells in the bottom channel, separated by a porous PDMS membrane.

To address the impact of 2-AA on endothelial cells, the first point of contact when the route of lung infection is through the bloodstream, we delivered 2-AA (20 µM) to the HPMEC channel and exposed the cells to continuous 2-AA flow for 20 hrs at a flow rate of 40 µl/h. In this setting, 2-AA can also reach the NHBE cells through the porous membrane. Continuous PBS flow in the non-2-AA-treated chips was used as a control. Following 20 h post-exposure to 2-AA or PBS, RNA was extracted from the cells of each channel individually, and RNA sequencing was performed. Principal component analysis (PCA) of the RNA-seq data revealed distinct differences between 2-AA-treated and PBS-treated cells, as well as between HPMEC and NHBE cells treated with 2-AA (**Supplementary Figures S1A, B**). Transcriptomic profiling revealed that 2-AA significantly impacts the transcription of multiple genes in both endothelial and epithelial cells, with altered expression of 4,499 genes in HPMEC and 623 genes in NHBE cells. In HPMEC, 2,558 genes were upregulated, and 1,941 genes were downregulated (**Figure 1B**), whereas in NHBE cells, 303 genes were upregulated, and 320 genes were downregulated (**Figure 1C**). Similarly to our previous study, which tested several types of cell lines (19, 21), increasing the concentration of 2-AA (20–200 μM) does not affect the cell viability of HPMEC cells (**Supplementary Figure S6**).

### 2-AA modulates multiple signaling pathways in HPMEC cells

We conducted a pathway enrichment analysis to identify the pathways modulated by 2-AA. The pathways predicted by Reactome were organized according to their statistical significance and assessed through p-values and false discovery rates (FDR). Analyses of upregulated and downregulated genes, as well as associated pathways, in HPMEC cells, are presented in **Supplementary Table S1**. Specifically, analysis of the 2,558 upregulated genes in these cells revealed 30 enriched pathways, primarily linked to a pro-inflammatory response, including interleukins (IL-17, IL-26, IL-3, IL-7), MAPK1/MAPK3 signaling, VEGFA-VEGFR2 signaling, and RUNX1-mediated transcriptional regulation (**Figure 1D**) (48). Other pathways involved with upregulated inflammatory responses were associated with the IL-6, TGF-β, TNF, and FoxO signaling, as well as metabolic responses, including the PI3K-Akt and receptor tyrosine kinase pathways (**Figure 1D**). Concurrently, pathways involved with anti-inflammatory responses, IL-10, IL-4, and IL-13 signaling were also upregulated (**Figure 1D**). In addition, G protein-coupled (GPCR) signaling, known for its contribution to modulating inflammatory cascades and influencing neutrophil degranulation, both critical components of innate immune response (49), was significantly upregulated.

To determine whether transcriptional modulation was accompanied by significant changes in the release of proinflammatory and anti-inflammatory cytokines, we measured cytokine levels in cell culture supernatants of the endothelial cells using the Bio-Plex Human Essential Immune Response Panel (13-plex) (**Supplementary Figure S2**). The levels of two pro-inflammatory cytokines, IL-6 and TNF-α (**Supplementary Figures S2A, B**) were significantly reduced relative to control cells in the supernatant of the 2-AA-treated endothelial cells (**Figure 1D**) despite the observed upregulation of their associated transcripts and regulatory genes (**Supplementary Table S1**). Notably, two key regulators of IL-6 secretion, *TIMP1* and *SOCS3*, along with six genes involved in the regulation and secretion of TNF (*TNFAIP3, CEBPB, BIRC3, SOCS3, and FRMD8*) were also upregulated (**Supplementary Table S1**). However, the levels of the anti-inflammatory cytokines IL-4 and IL-10 showed no significant difference in the supernatant of the endothelial cells (**Supplementary Figures S2C, D**), although their gene expression was also found to be upregulated (**Figure 1D**). These findings suggest that 2-AA treatment may exert anti-inflammatory effects by modulating cytokine secretion at a post-transcriptional level, potentially contributing to an overall reduction in endothelial inflammatory responses.

Additional upregulated pathways include the estrogen receptor (ESR) signaling pathway, nuclear transcription pathway, Hypoxia-inducible factor 1 (HIF-1) signaling pathway, and vitamin D receptor pathway (**Figure 1D**). Importantly, our analysis revealed the upregulation of histone genes and pathways related to the cellular response to stress and degradation of the extracellular matrix, including the expression of matrix metalloproteinases (MMPs) such as *MMP9 MMP15*, *MMP19*, *MMP25*, and *MMP3*, as well as keratinization and glycosaminoglycan metabolism (**Supplementary Table S1**).

Pathway analysis of 1941 downregulated genes in HPMEC cells identified 16 pathways predominantly linked to interferon-alpha/beta/gamma signaling and IL-1 signaling, contributing to decreased inflammatory responses. Along these lines, Toll-like receptor signaling and p53 signaling were downregulated (**Figure 1E**), a result that might be associated with prolonged (20 hrs) rather than acute exposure to 2-AA. Similarly, 2-AA was found to downregulate the biosynthesis of nucleotide sugars, which are crucial for glycosylation, nicotinate metabolism, and metabolism of water-soluble vitamins and cofactors. In addition, 2-AA leads to a downregulation of oxidative phosphorylation, potentially resulting in mitochondrial dysfunction and an increased production of reactive oxygen species (ROS), which further influences inflammation and the cell stress response (1, 32). Notably, 2-AA downregulates the metabolism of important homeostatic molecules, including steroids, cholesterol, fatty acids, retinoic acid synthesis, tryptophan metabolism, and phosphatidylinositol signaling (**Figure 1E**). These findings underscore the significant role of 2-AA in modulating the transcription of multiple genes and pathways that govern endothelial function (**Figure 1D**).

### Pathway responses to 2-AA exposure in NHBE cells

The Reactome pathway prediction of the effect of 2-AA on bronchial airway epithelium responses revealed 12 significant pathways and 303 upregulated genes. These pathways were primarily linked to pro-inflammatory responses, such as IL-12 and VEGFA-VEGFR2 signaling, as well as anti-inflammatory responses, including IL-4 and IL-13 signaling (**Figure 1F**). Furthermore, pathways related to neutrophil degranulation, cellular response to stress, and keratinization, which are associated with cell survival and differentiation, were also upregulated. Notably, and in contrast to the effects on endothelial cells described above, the mitochondrial oxidative phosphorylation pathway exhibited upregulation, as well as other pathways related to the vitamin D receptor pathway and metabolism. The latter included pathways associated explicitly with steroid, cholesterol, lipid, and glycolipid metabolism (**Figure 1F**). These findings may reflect the distinct roles of the endothelium and epithelium in responding to exposure to 2-AA and the progression to the chronic phase of the associated infection.

In contrast, the analysis of the 320 genes that underwent downregulation in NHBE cells revealed significant enrichment in 10 pathways predominantly associated with antibacterial and antiviral responses. These responses were mediated by interferon-alpha/beta/gamma signaling and negative regulators of the DDX58/IFIH1 signaling pathway. Additionally, we observed the downregulation of pathways related to toll-like receptor signaling, which is associated with the early innate immune response and TNF signaling (**Figure 1G**). Also downregulated were pathways related to signaling by receptor tyrosine kinases involved in cell differentiation and the degradation of the extracellular matrix-associated genes crucial for remodeling (50). Notably, the downregulation was extended to pathways involved in nucleotide, steroid, and retinol metabolism (**Figure 1G**). The analysis of both upregulated and downregulated genes, along with their associated pathways, for NHBE cells is presented in **Supplementary Table S2** and reflects the altered inflammatory response.

These results indicate that exposure to the quorum-sensing molecule 2-AA induces extensive transcriptional changes in lung endothelial and epithelial cells. The distinct response observed in the two different cell types implies a complex interaction between the bacterial signaling molecule and the specific cellular mechanisms in these cells.

### 2-AA modulates MMP-9 protein levels in endothelial cells

**Figure 2:**
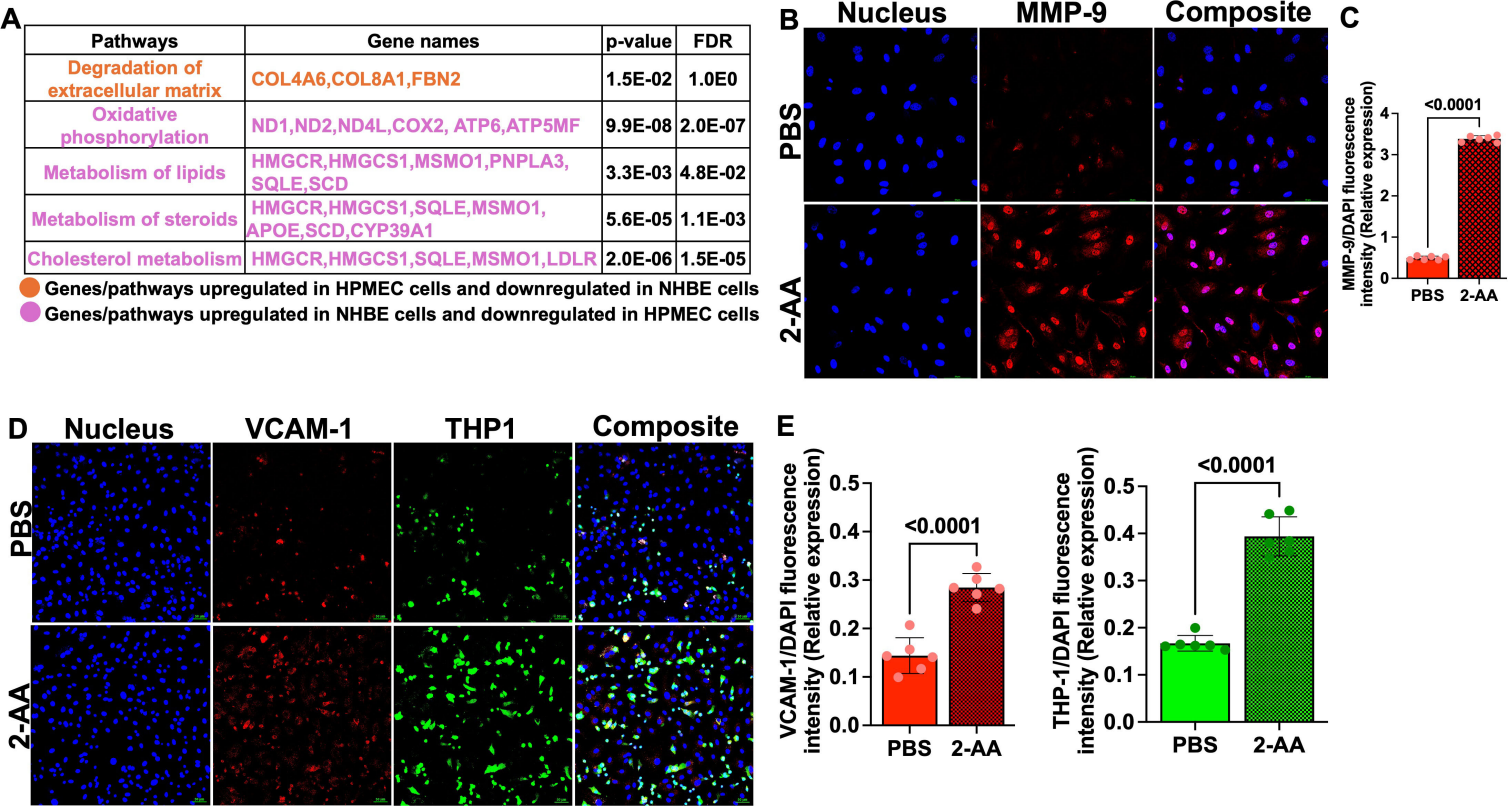
2-AA upregulates MMP-9 and VCAM-1 levels and enhances THP-1 monocyte adhesion in HPMECs. **(A)** Table depicting the differentially expressed genes and their associated pathways in HPMEC and NHBE cells following 2-AA treatment in the airway-on-a-chip. Genes and pathways upregulated in HPMEC cells and downregulated in NHBE cells (orange). Genes and pathways upregulated in NHBE cells and downregulated in HPMEC (purple). Significance is denoted by p<0.05 and FDR<0.05. **(B)** Representative confocal images of MMP-9 (red) in 2-AA-treated and untreated HPMEC cells. Nuclei were counterstained with DAPI. **(C)** Relative expression of MMP-9 fluorescence intensity normalized to DAPI. **(D)** Representative immunofluorescence images of VCAM-1 (red) and adherent THP-1 monocytes (green) in 2-AA-treated and untreated HPMEC cells. **(E)** Quantification of VCAM-1 and THP-1 fluorescence intensity normalized to DAPI. Composite images show all channels merged. The scale bar represents 50 μM. The images are representative of n=3 independent experiments, with six images analyzed per group. Each dot represents six different biological replicates (n = 6), error bars denote mean ± standard deviation. P-values were calculated using a two-tailed unpaired t-test, depicted on the graphs. The overall p-value summary is p < 0.0001, indicating statistical significance.

In HPMEC cells, we found upregulation of genes associated with the degradation of extracellular matrix (*COL4A6, COL8A1, FBN2*) by AA, indicating potential alterations in endothelial barrier integrity. In contrast, in NHBE cells, these genes exhibited downregulation, reflecting differing cellular responses that may influence barrier permeability and subsequent cellular functions in the epithelium (**Figure 2A**).

MMP-9 is a key component in the maintenance of the integrity of cellular barriers (51), we assessed MMP-9 expression in HPMECs following 2-AA treatment by immunofluorescence staining. Confocal microscopy of HPMECs cells treated with 2-AA and stained with anti-MMP-9 antibody revealed a ~3-fold increase in MMP-9 abundance relative to untreated cells (**Figures 2B, C**). The cellular distribution pattern of MMP-9 in 2-AA-treated cells appeared to be predominantly cytoplasmic with some perinuclear localization, which is consistent with its known subcellular distribution and role in extracellular matrix remodeling (52). The increase in MMP-9 protein levels supports the transcriptomic data, and suggest that 2-AA treatment compromises tight junction integrity of the endothelial cells.

### 2-AA promotes aberrant monocyte adhesion to endothelial cells

VCAM-1 expression triggered by stress response can induce monocyte adhesion and cell migration (53). We determined the impact of 2-AA on VCAM-1 expression and THP-1 monocyte adhesion to microvascular endothelial cell monolayers. HPMECs treated with 2-AA exhibited markedly increased VCAM-1 red fluorescence across the endothelial cell surface relative to control (**Figure 2D**). The adhesion of THP-1 monocytes (green fluorescence) were also significantly enhanced by 2-AA (**Figure 2D**). The composite images support increased colocalization (appearing as yellowish-white regions) between VCAM-1 expression and adhered THP-1 cells in the 2-AA condition. Quantification shows a ~2-fold increase in the abundance of VCAM-1 and a ~2.2-fold increase in the abundance of THP-1 cell adherence to 2-AA-stimulated endothelial monolayers compared to the PBS control (**Figure 2E**). These findings indicate that 2-AA promotes endothelial activation, as evidenced by the upregulation of VCAM-1, which consequently facilitates increased adhesion of immune cells.

### Insights into the common and different pathways and genes regulated by 2-AA in HPMEC and NHBE cells

**Figure 3:**
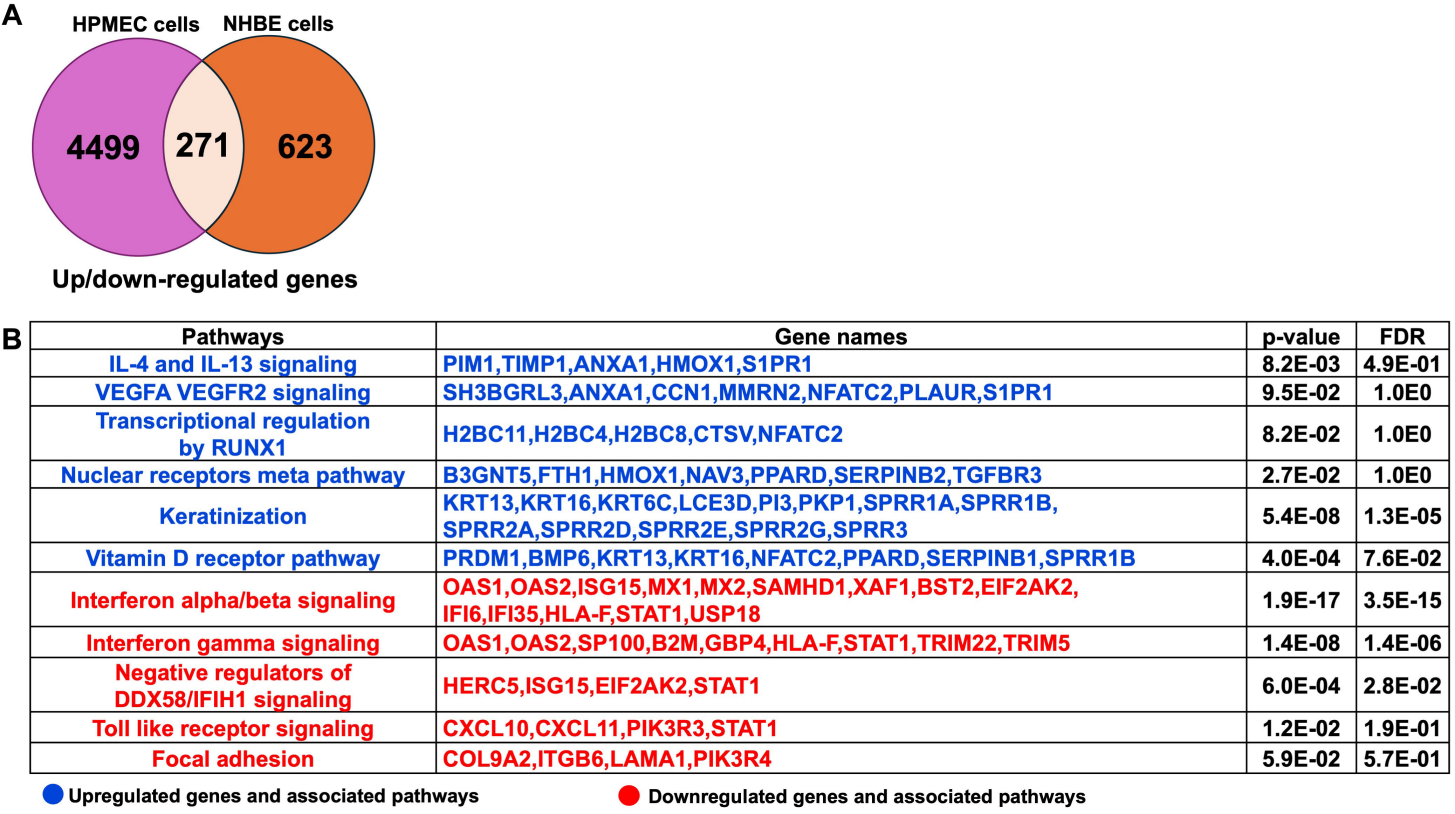
Common genes and associated pathways regulated by 2-AA in HPMEC and NHBE cells. **(A)** A Venn diagram and table depict gene expression data. The Venn diagram shows 4,499 genes up- or down-regulated in HPMEC cells, 623 in NHBE cells, and 271 that are overlapping. **(B)** Upregulated (blue) and downregulated (red) genes and pathways, common in NHBE and HPMEC cells with a p-value < 0.05 and a False Discovery Rate (FDR) < 0.05. The DAVID web server was used for the Reactome pathway enrichment analysis.

The comprehensive pathway analysis of 2-AA-treated HPMEC and NHBE cells prompted us to identify the common genes and their associated pathways that are significantly regulated by both HPMEC and NHBE cells in response to 2-AA. Upon comparing the 4,499 genes from HPMEC and the 623 genes from NHBE cells (**Supplementary Table S2**), we identified a total of 271 genes that were common and significantly regulated in both HPMEC and NHBE cells (**Figure 3A**). A pathway enrichment analysis to determine the pathways and genes that are either upregulated or downregulated identified the following genes as being upregulated by 2-AA: *PIM1*, *TIMP1*, *ANXA1*, *HMOX1*, and *S1PR1*. These genes are interconnected with the IL-4 and IL-13 signaling pathway, promoting an anti-inflammatory response. Additionally, genes such as *SH3BGRL3, ANXA1, CCN1, MMRN2, NFATC2, PLAUR, and SIPR1, which are* associated with the pro-inflammatory response and increased endothelial cell permeability via VEGFA-VEGFR2 signaling, were also upregulated. Furthermore, genes *H2BC11, H2BC4, H2BC8, CTSV, and NFATC2, which RUNX1 regulates*, were upregulated, contributing to cell differentiation and innate immunity. Genes related to keratinization, including *KRT13, KRT16, KRT6C, LCE3D, PI3, PKP1, SPRR1A, SPRR1B, SPRR2A, SPRR2D, SPRR2E, SPRR2G, SPRR3* were similarly upregulated. Other notable upregulated genes included *B3GNT5, FTH1, HMOX1, NAV3, PPARD, SERPINB2*, and *TGFBR3* which are related to the nuclear receptors meta pathway as well as *PRDM1, BMP6, KRT13, KRT16, NFATC2, PPARD, SERPINB1, and SPRR1B* associated with the Vitamin D receptor pathway (**Figure 3B**).

Conversely, the following pathways and genes were downregulated in both cell types: those involved in interferon-alpha/beta signaling (*OAS1, OAS2, ISG15, MX1, MX2, SAMHD1, XAF1, BST2, EIF2AK2, IFI6, IFI35, HLA-F, STAT1, USP18*) and negative regulators of DDX58/IFIH1 signaling (*HERC5, ISG15, EIF2AK2, STAT1*) which are associated with antiviral responses. Additionally, genes related to interferon-gamma signaling (*OAS1, OAS2, SP100, B2M, GBP4, HLA-F, STAT1, TRIM22, TRIM5*), which are associated with antibacterial responses, were also downregulated in both cell types. The Toll-like receptor signaling pathway and downstream genes, including *CXCL10, CXCL11, PIK3R3, and STAT1*, related to the innate immune response were similarly downregulated. Furthermore, genes such *as COL9A2, ITGB6, LAMA1*, and *PIK3R4*, which regulate the focal adhesion pathway, were downregulated, indicating a potential impact on decreased cell migration and proliferation, as well as impaired tissue repair processes (**Figure 3B**).

Next, genes and associated pathways specifically expressed in HPMEC or NHBE cells in response to 2-AA are shown in **Supplementary Table S2**. The pathways and genes related to the pro-inflammatory IL-12 signaling, antiviral response via interferon-alpha/beta signaling, keratinocyte differentiation, steroid metabolism, glycosphingolipid biosynthesis, and pyrimidine metabolism were differentially regulated in NHBE cells (**Supplementary Table S2A**). In contrast, the pathways specific to HPMEC cells included signaling pathways regulated by IL-17, IL-3, IL-7, IL-1, IL-18, IL-6, TLR4, IL-10, IL-4, IL-13, TNF, TGFB, FOXO, VEGFA-VEGFR2, PI3-Akt, NF-kappa B, MAPK, receptor tyrosine kinase, ESR, p53, HIF-1 and neutrophil degranulation. Pathways related to lung fibrosis, cellular response to stress, degradation of extracellular matrix, and glycosaminoglycan biosynthesis were also affected by 2-AA in HPMEC cells. Additionally, pathways associated with retinol metabolism, fatty acid metabolism, and phosphatidylinositol signaling were also influenced by the 2-AA treatment in HPMEC cells (**Supplementary Table S2B**). Thus, the 2-AA response on HPMEC and NHBE cells underscores the importance of cellular context and experimental setting in determining the potential outcome of inflammatory responses and metabolic processes *in vivo*.

### 2-AA modulates the expression of genes associated with mitochondrial function and cholesterol metabolism in human lung cells

**Figure 4:**
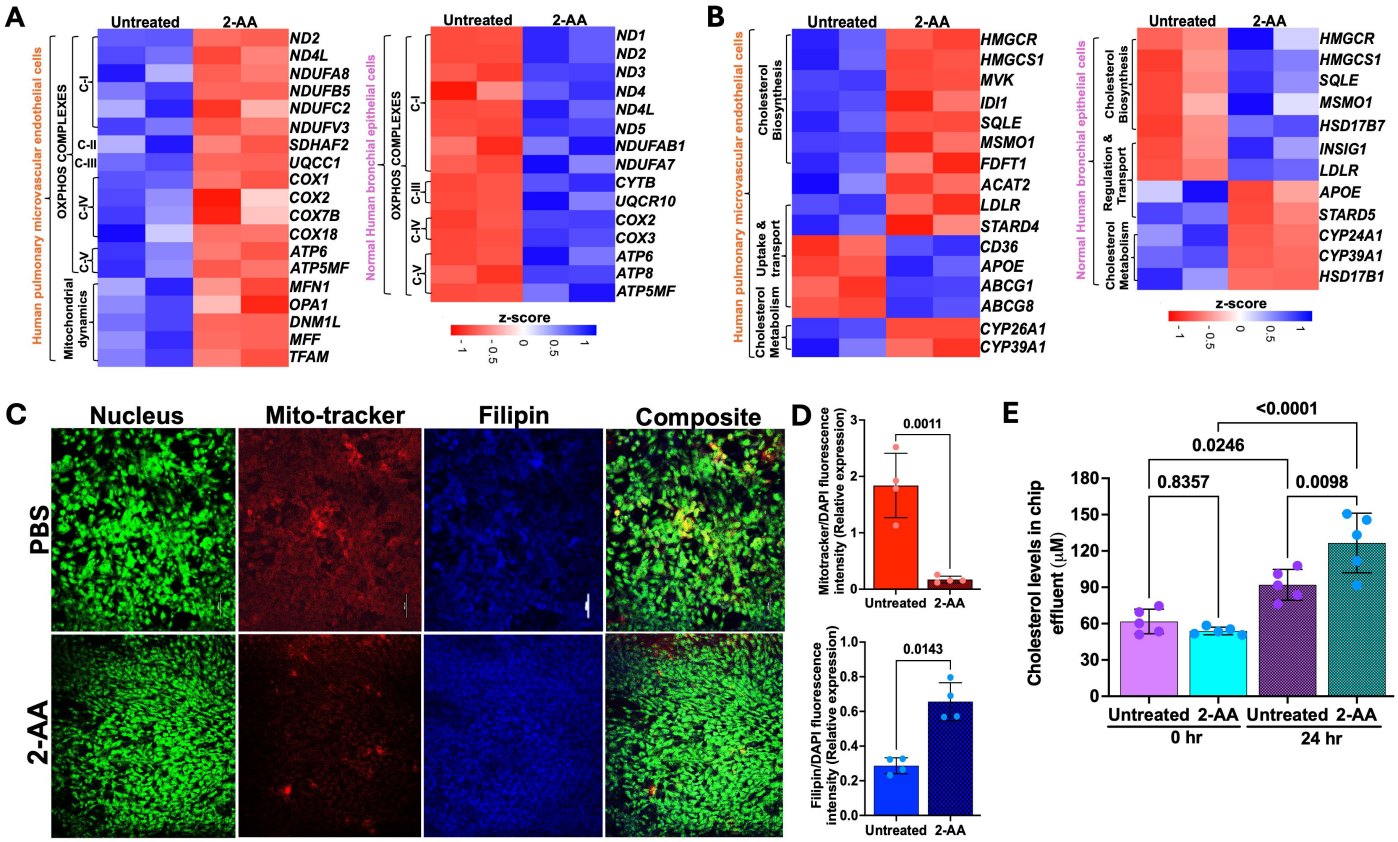
2-AA differentially regulates the mitochondrial and metabolic functions in HPMEC and NHBE cells. Heatmaps showing the differentially expressed genes in HPMEC and NHBE cells treated with PBS or 2-AA **(A)** for mitochondrial OXPHOS and mitochondrial dynamics, and **(B)** for cholesterol biosynthesis, uptake, transport, and metabolism. The z-score represents the upregulated (blue) and downregulated (red) genes. **(C)** Fluorescence microscopy and quantification showing effects on cholesterol in different cell types with PBS and 2-AA treatments. Representative confocal images showing total cholesterol staining with Filipin (blue) and mitochondrial respiration staining with Mitotracker (red) in chips (n=2). Nuclei were counter-stained with DAPI. The scale bar represents 50 μM. **(D)** Relative expression depicting the intensity of mitotracker (red) versus intensity of DAPI and intensity of Filipin (blue) to DAPI. **(E)** Amplex red quantification of total cholesterol from the effluent of the airway-on-a-chip cells treated with PBS or 2-AA at 0 and 24 hours. In panel **(D)**, each dot represents n=4 independent experiments; and in panel E n=5 different biological replicates. Error bars denote mean ± standard deviation. In panel **(D)**, p-values were calculated using a two-tailed unpaired t-test. In panel **(E)**, p-values were calculated using ordinary one-way ANOVA followed by Tukey’s post-test. Exact p-values are depicted on the graphs. The overall p-value summary for panels E and F was p < 0.01, indicating statistical significance.

Several of the 271 genes identified as common were differentially expressed in the HPMEC versus NHBE cells. 2-AA has been shown to disrupt key mitochondrial functions in skeletal muscle and affect the TCA cycle in macrophages, leading to increased oxidative stress and decreased energy production (2, 32). In HPMEC cells, we also found that 2-AA downregulates mitochondrial functions. Genes associated with mitochondrial OXPHOS complex (*ND1*, *ND2, ND4L,COX2, ATP6, ATP5MF*) were downregulated (**Figure 2A**). The heatmap analysis further emphasizes this effect by showing a consistent decline in the expression of genes encoding mitochondrial complexes I-V alongside essential genes that manage mitochondrial dynamics, such as *MFN1, OPA1, DNM1L, MFF*, and *TFAM* (**Figure 4A**).

Conversely, NHBE cells exhibited a distinct response pattern, with genes associated with the mitochondrial OXPHOS complex being upregulated in response to 2-AA (**Figure 4A**). This suggests an adaptive or compensatory mechanism whereby NHBE cells enhance mitochondrial function in the presence of 2-AA, an additional mechanism of the mounting inflammatory response of the epithelium. The ability of NHBE cells to upregulate mitochondrial complex I and complexes III-V indicates changes in metabolic capability relative to HPMEC cells. This could be linked to differences in cell types, stress responses, or inherent metabolic capacities triggered by inflammation between these cell types.

Indeed, we observed contrasting gene expression patterns relating to lipid, steroid, and cholesterol metabolism in NHBE and HPMEC cells. Notably, NHBE cells exhibited a significant upregulation of several key genes, including *HMGCR, HMGCS1*, *SQLE, MSMO1, APOE, LDLR, SCD, CYP39A1, PNPLA3*, which are integral to lipid biosynthesis and/or storage and cholesterol metabolism (**Figure 2A**). This indicates lipid synthesis in response to 2-AA in the NHBE cells. In contrast, HPMEC cells downregulated similar cholesterol biosynthesis genes, specifically *HMGCR*, *HMGCS1, MVK, IDI1, SQLE, MSMO1, and FDFT1* (**Figure 4B**). This reduction suggests a compromised lipid metabolic pathway, impairing cholesterol biosynthesis in the HPMEC cells. In line with the above, we observed a differential regulation of cholesterol uptake and efflux genes. HPMEC cells responded by upregulating *CD36, APOE*, and efflux genes *ABCG1* and *ABCG8*, in contrast to the increase in *LDLR* gene expression observed in NHBE cells (**Figure 4B**). This suggests cell-type-specific alterations in cholesterol biosynthesis, transport, and utilization, supporting cell-specific modulation of energy utilization by 2-AA. However, both cell types exhibited downregulation of cytochrome P450 family genes, which are also involved in downstream cholesterol metabolism, specifically *CYP26A1, CYP39A1, CYP24A1, and HSD17B1*, which catalyzes the reduction of estrone to the most potent estrogen, estradiol, possibly altering receptor signaling (**Figure 4B**) (54).

### 2-AA induces mitochondrial dysfunction in HPMEC cells and cholesterol accumulation in NHBE cells

Based on the 2-AA-mediated differential gene expression of mitochondrial respiration-related genes and cholesterol biosynthesis, uptake, and efflux genes, we stained 2-AA- and PBS-treated chips with the Mitotracker dye to label mitochondria and filipin dye to assess cholesterol abundance in respiring cells (**Figure 4C**). Fluorescence confocal microscopy images of the stained 2-AA lung-on-chip showed significantly reduced mitochondria staining compared to the PBS-treated control chip, supporting the dysfunction in the bioenergetic capacity suggested in the transcriptomic studies (**Figures 4C, D**). In contrast, accumulation of cholesterol was observed in the 2-AA-treated cells compared to the PBS-treated chips (**Figures 4C, D**). Assessment of extracellular cholesterol in the effluent collected from the airway-on-a-chip HPMEC channel before and after 24 hrs of 2-AA or PBS treatment reveals a significant increase in extracellular cholesterol levels at 24 hrs compared to control PBS-treated samples (**Figure 4E**). These findings indicate a direct correlation between mitochondrial dysfunction and metabolic disturbances.

**Figure 5:**
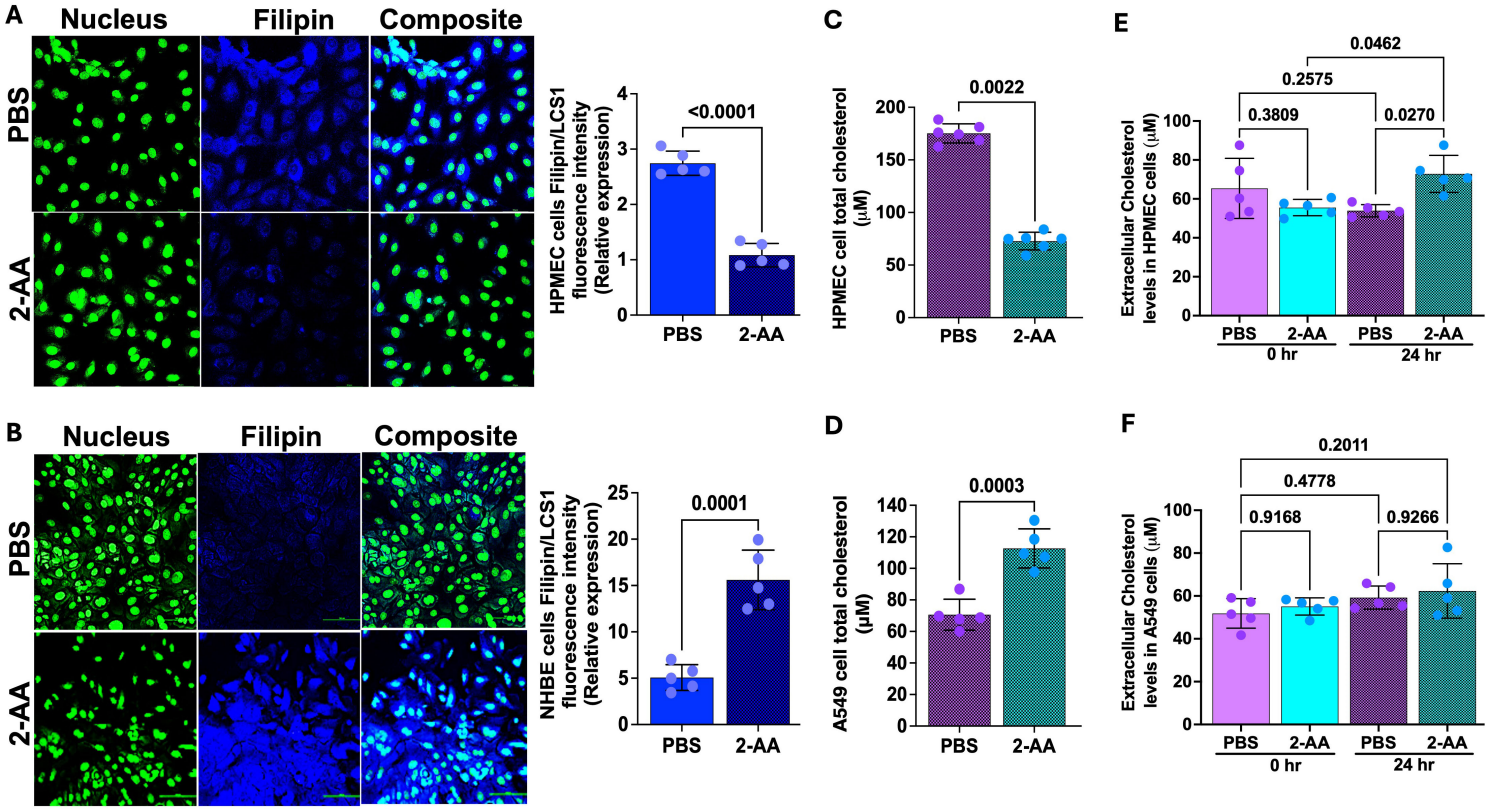
2-AA promotes cholesterol accumulation in epithelial cells. **(A, B)** Representative confocal images and relative fluorescence intensities in six random fields of HPMEC and NHBE cells in chamber slides treated with 2-AA or PBS. Cells were stained with Filipin (blue), and nuclei (green) with LCS1. Scale bars represent 20μm. **(C, D)** Amplex red quantification of total cell cholesterol of HPMEC **(C)** or A549 **(D)** cells post-2-AA treatment or PBS in cell culture plates. **(E, F)** Amplex red quantification of total cholesterol in the supernatant HPMEC **(E)** or A549 **(F)** cells at 0 and 24 hours, treated with 2-AA or PBS. Each dot represents the mean of n= 5 biological replicates; error bars denote the standard deviation. In panel **(A)**, p-values were calculated by a two-tailed unpaired t-test, and in panel **(E, F)**, ordinary one-way ANOVA followed by Tukey’s post-test was used. Exact p-values are depicted on the graphs, with an overall p-value summary for the panels **(A–D)** is p < 0.0001, and panels **(E, F)** is p < 0.01, indicating significance.

Cholesterol represents 5-10% of the lipid component of lung surfactants, playing a vital role in reducing surface tension (55, 56) at the air-liquid interface of the alveoli. The staining results from the lung-on-chip platform cannot differentiate whether the 2-AA-mediated accumulation of cholesterol occurs in the epithelial or endothelial cells. Thus, to determine the cell type associated with the increase in cholesterol observed and correlate the findings with the transcriptome data from the chip suggesting an increase in cholesterol in the epithelial cells, we used chamber slides and stained separately epithelial and endothelial cells, together with evaluation of the cholesterol levels in total cell lysates (**Figure 5**). First, using the same primary human HPMEC and NHBE cells and fluorescence confocal microscopy, we observed a marked reduction in Filipin staining in HPMECs treated with 2-AA compared to those treated with PBS (**Figure 5A**), reflecting a decrease in cellular cholesterol levels in the 2-AA-treated cells. Consistent with the microscopy data, quantification of the total cellular cholesterol in 2-AA treated HPMECs cell lysates shows significantly lower amounts of cholesterol relative to PBS-treated controls (**Figure 5C**). Moreover, the extracellular cholesterol levels in the supernatant of HPMECs, measured before and after 24-hour treatment with 2-AA, showed a significant increase at 24 hrs compared to controls (**Figure 5E**), suggesting alterations in cholesterol synthesis, uptake, or efflux mechanisms due to 2-AA exposure. In contrast, fluorescence confocal microscopy (**Figure 5B**) of the 2-AA-treated NHBE cells stained with Filipin showed an increased intensity of Filipin relative to the PBS controls. To further confirm this finding in other epithelial cells, we measured the total cholesterol content in the human lung epithelial cell line A549, a cell line broadly used in preclinical research (**Figure 5D**). After 24 hrs of 2-AA exposure, A549 cells also demonstrated significantly elevated cholesterol levels compared to PBS-treated cells (**Figure 5D**). Extracellular cholesterol measurement from the supernatant of these cells, collected before and after 24 hrs of treatment with 2-AA, revealed no significant changes in extracellular cholesterol levels at either time point or when compared to the PBS-treated (control) group. These results support the notion that while HPMEC cells actively synthesize and efflux cholesterol, epithelial cells synthesize and accumulate it at extracellular levels; they did not result in measurable changes between 0 and 24 hrs (**Figure 5F**). Further support for these findings is the increase in *HMGCR* gene expression in epithelial cells (**Figure 4B**) in the lung-on-chip, as well as the protein levels of HMGCR observed in murine lung section of 2-AA-treated mice stained with the anti-HMGCR antibody against the rate-limiting enzyme HMG-CoA reductase (**Supplementary Figure S4A**). We observed that 2-AA-treated lung tissues exhibited a thicker epithelial cell layer compared to the untreated lung tissues (**Supplementary Figure S4A**). This finding could be associated with cholesterol accumulation in the lung tissues of 2-AA-treated mice. Moreover, since excessive accumulation of cholesterol results in lung inflammation and fibrosis, we performed a haematoxylin and eosin staining (H&E) to investigate whether enhanced cholesterol levels in the lungs are associated with significant lung fibrotic remodeling. **Supplementary Figure S4B** shows a progressive thickening of the bronchiolar and alveolar walls, indicating structural alterations in lung architecture in the 2-AA-treated versus untreated lung tissues.

### 2-AA modulates the expression of cystic fibrosis and idiopathic pulmonary fibrosis-related genes in both HPMEC and NHBE cells

**Figure 6:**
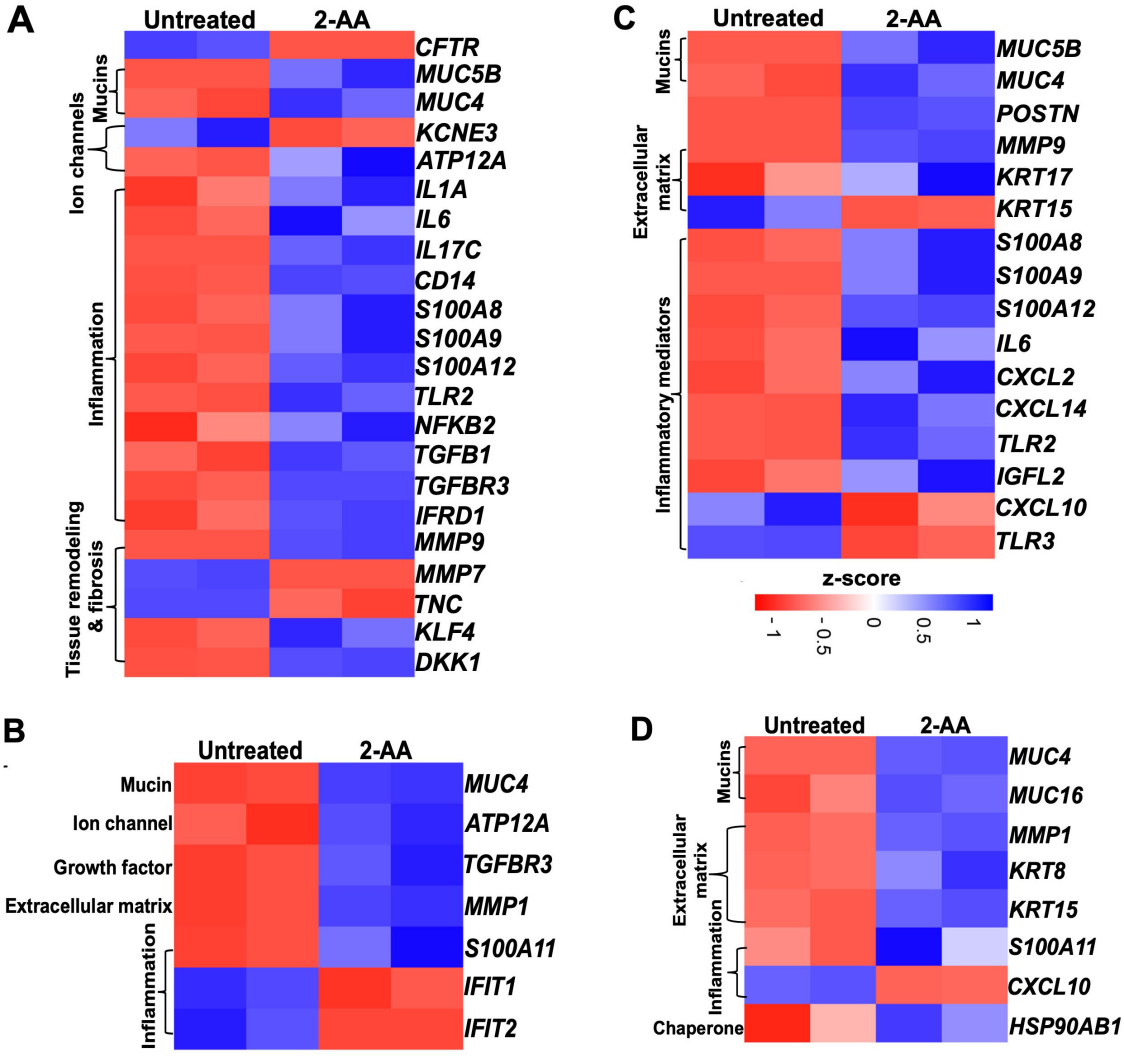
Airway-on-a-chip studies reveal the impact of 2-AA on the expression of cystic fibrosis and idiopathic pulmonary fibrosis-related genes in HPMEC and NHBE healthy cells. Heatmaps show gene expression differences between untreated and 2-AA-treated conditions. Panels show the gene markers associated with **(A, B)** cystic fibrosis and **(C, D)** with idiopathic pulmonary fibrosis in healthy HPMEC and NHBE cells. The z-score color range represents the upregulated (blue) and downregulated (red) genes.

Cystic fibrosis (57) and idiopathic pulmonary fibrosis (IPF) are complex genetic disorders characterized by chronic inflammatory changes in the epithelial cells, which affect lung functions (58, 59). Our findings indicate that 2-AA significantly modulates the expression of genes associated with CF (**Figures 6A, B** and **Supplementary Figure S5**) and IPF (**Figures 6C, D**) in both HPMEC and NHBE cells. Heatmap analysis revealed that *in* HPMEC cells, 2-AA led to the downregulation of the cystic fibrosis gene marker *CFTR*, which is essential for bicarbonate secretion, while upregulating *KLF4*. This transcription factor can directly suppress the expression of the *CFTR* gene (60). HPMEC and NHBE cells exhibited upregulation of the mucin genes, specifically *MUC5B* and *MUC4*, contributing to mucus accumulation in the lungs (**Figures 6A, B**). These genes were also associated with IPF (**Figure 6C**). Additionally, *KCNE3*, a voltage-gated potassium channel critical for normal chloride ion transport (61), was downregulated in response to 2-AA in endothelial cells. At the same time, the H+/K+-ATPase *ATP12A* (62), associated with airway surface liquid (63) and acidification, was upregulated in both cell types.

The treatment with 2-AA also induced an unbalanced cytokine response in endothelial cells, with upregulation of pro-inflammatory cytokines (64–66) such as *IL-1α*, *IL-6*, *IL-17c*, and *NFKB2*, which are known to amplify inflammation and innate immune responses (67, 68), including *CD14* and *TLR2* (**Figures 6A, B**). Of these, *IL-6 and TLR2*, associated with IPF, were also found to be upregulated with 2-AA treatment (**Figure 6C**). Furthermore, the *S100* calcium-binding family gene markers for both CF and IPF, which act as chemokines for neutrophil recruitment to sites of inflammation (69–71), were upregulated, including *S100A8*, *S100A9* and *S100A12* in endothelial cells, and *S100A11* in epithelial cells. Notably, the Wnt and TGFβ signaling pathways have been identified as crucial players in maintaining Iung epithelial polarization and differentiation in the airways. At the same time, TGFβ is also a primary driver of fibrosis (72). These proteins also play a crucial role in maintaining and regulating the tissue matrix’s homeostasis. Thus, the upregulation of *TGFB1* and *TGFBR3*, alongside 2-AA treatment, suggests increased fibrotic burdens (73), while *DKK1*, a known Wnt antagonist, indicates potential dysregulation of the Wnt signaling pathway (72). Additionally, *IFRD1* (Interferon-related developmental regulator 1), identified as a modifier of cystic fibrosis lung disease severity, was also upregulated in endothelial cells (74). MMPs (matrix metalloproteases), which are implicated in extracellular matrix turnover, tissue degradation, and repair, exhibited upregulation of *MMP-9*, while *MMP-7* (75, 76) was downregulated in endothelial cells and MMP-1 in epithelial cells (77). These changes indicate that the membrane architecture and tissue homeostasis are dysregulated.

In the adjacent NHBE cells (**Figure 6B**), in addition to the CF gene markers (*MUC4*, the H+/K+ ATPase *ATP12A*, and the inflammatory *TGFBR3*, which mirror the expression observed in HPMEC cells, other genes were found to be expressed specifically in these epithelial cells. The matrix metalloprotease *MMP1*, which plays a role in degrading extracellular matrix components (78), was upregulated in conjunction with *S100A11*, another calcium-binding gene (79). Conversely, *IFIT1* and *IFIT2* (80), which are associated with the interferon response, were downregulated, potentially increasing susceptibility to bacterial infection. The observed dysregulation of inflammation and mucus production by 2-AA suggests a potential role of this signaling molecule in exacerbating lung fibrosis in CF.

Some additional IPF marker genes were upregulated in 2-AA-treated HPMEC cells, including *POSTN* (periostin) and *KRT17*, both of which contribute to extracellular matrix production. Conversely, *KRT15* expression was downregulated, which may contribute to the compromised clearance of damaged cells and consequent triggering of fibrosis (81) (**Figure 6C**). Furthermore, pro-inflammatory chemokines *CXCL2* and *CXCL14* (82, 83) were upregulated, while *TLR3* and *CXCL10*, associated with anti-fibrotic properties, were downregulated. Additionally, the expression of *IGFL2* (insulin-like growth factor-like family member 2), secreted from the extracellular matrix, was also increased (84).

Analysis of the IPF gene markers in the adjacent NHBE cells revealed a response to 2-AA treatment comparable to that seen in HPMEC cells, with upregulation of keratin *KRT15* and downregulation of chemokine *CXCL10* (**Figure 6D**). Genes were found to be upregulated only in NHBE cells. However, some of the functions found to be affected in HPMEC cells included keratin *KRT8* (85), *MUC16, which is* implicated in fibrotic processes (86) through its interaction with TGFB1 and the canonical pathways, as well as the cellular senescence-associated gene *HSP90AB1* (87). These results suggest that 2-AA plays a role in promoting pathological changes associated with lung fibrosis in endothelial and epithelial cells.

## Discussion

In this study, using an cutting-edge technology that mimics the organotypic microenvironment of the human airway, we uncovered multiple common and cell-specific 2-AA-mediated responses, underscoring the multifaceted role of this *PA* QS-secreted small molecule on pulmonary function and physiology. The platform’s emulation of the human hemodynamic environment and physiological biomechanics, as established by flow and tissue-relevant stretching, enabled the evaluation of the impact of this MvfR-regulated QS small molecule in a dynamic and physiologically relevant human environment constructed by pulmonary microvascular endothelial cells and bronchial epithelial cells.

The multifaceted effects of 2-AA align with this study’s findings, as the HPMEC and its adjacent NHBE cells exhibit multiple common and cell-specific responses to 2-AA, affecting various signaling pathways and genes. While the endothelial and bronchial epithelial cells are not in direct contact in the lungs, their crosstalk through paracrine signals, inflammation, and shared microenvironment changes play a critical role in regulating lung function during health and disease (88, 89). The microvascular endothelium may secrete a variety of soluble mediators that can diffuse to the epithelium and affect their function (90), promoting paracrine signaling between the two cell types. The setup of the chip with the two cell types settled in separate channels offers the possibility to decipher cell-cell communication, and the overall impact of 2-AA on the gene responses of endothelial and epithelial cells.

Analyses of the transcriptomic findings delineated common and distinct physiological changes in epithelial and endothelial cells. We found that 2-AA in HPMEC cells affects key innate immune response pathways by impacting inflammatory cytokines. 2-AA treatment significantly reduced the presence of IL-6 and TNF-α in the HPMEC cell supernatant. The IL-6 and TNF-α reduction is particularly significant given the established roles of these cytokines in endothelial dysfunction and vascular inflammation (91–93). The upregulation of *ZC3H12A* (also known as Regnase-1), a known post-transcriptional regulator that degrades *IL-6* and *TNF-α* mRNA, may explain the observed discrepancy between increased transcript levels and reduced *IL-6* and TNF-α cytokines in the cell supernatant. They suggest that 2-AA modulates inflammatory responses post-transcriptionally and potentially exerts a protective effect against inflammation and injury.

The upregulation of extracellular matrix production in NHBE cells, and moreover, that of the matrix metalloproteinases in HPMEC cells, including the increased protein levels of MMP-9 suggests that this molecule contributes to extracellular matrix degradation and potential disruption of endothelial barrier integrity, which could facilitate bacterial invasion during infection. The increased disruption of barrier integrity often accompanies leukocyte adhesion and transmigration in tissues. Our results, show increased propensity of monocyte adherence to 2-AA treated endothelial cells, mediated by increased VCAM-1 expression. This observation aligns with the previously established role of these genes in maintaining the basement membrane structure, which is vital for maintaining endothelial cell barrier integrity (94–96).

Notably, 2-AA-treated HPMEC cells exhibit a compromised mitochondrial respiratory chain, impaired ATP production, and, consequently, a compromised energy metabolism within the cells. This aligns with the mitochondrial dysfunction we previously reported for this MvfR-regulated molecule (1, 2, 34) and observed in lung disorders (97). The downregulation of mitochondrial energy metabolism by 2-AA is expected to lead to functional impairments that adversely affect cellular energy homeostasis in HPMEC cells. Interestingly, while the associated pathways known to respond to hypoxic conditions, such as HIF-1 signaling, were upregulated in HPMEC cells, an opposite effect was observed in bronchial epithelial cells, suggesting either functional or hyperactive mitochondria. These results suggest that bacterial infection leads to increased aerobic glycolysis in infected bronchial epithelial cells (98) which are essential for the cells to survive the infection and maintain airway functionality.

Cholesterol is the major neutral lipid in pulmonary surfactant (99). It is well established that alterations in cholesterol metabolism and their implications play a fundamental role in pulmonary surfactant composition, thereby contributing to lung function (100). In a lung-on-chip model of murine alveoli, alveolar surfactant was shown to play a crucial role in inhibiting mycobacterial growth (101). Our results show significant alterations in cholesterol metabolism in both HPMEC and NHBE cells, a molecule with multifaceted functionalities in cellular processes. In HPMEC cells, we observed an upregulation of genes associated with cholesterol uptake, including *CD36*, *APOE*, and the efflux genes *ABCG1* and *ABCG8*, suggesting an enhanced requirement or capability for cholesterol utilization and storage in these cells. Conversely, the NHBE cells exhibited an increase in *LDLR* gene expression, which plays a crucial role in cholesterol uptake. This was further confirmed by the observed increase in extracellular cholesterol in the supernatant of HPMEC cells and the increase in intracellular cholesterol in NHBE cells following 2-AA treatment. The observed pattern implies a potential uptake of extracellular cholesterol by NHBE cells, possibly stemming from efflux mechanisms activated in HPMEC cells. These results suggest a potential crosstalk in cholesterol metabolism between these cells.

Altered cholesterol homeostasis has been reported to be involved in lung inflammation and injury (102), and its accumulation may interfere with the capacity of the lung surfactant, potentially exacerbating conditions such as pulmonary fibrosis. Our results from HMGCR staining of the lung tissue from mice treated with 2-AA show an accumulation of cholesterol in the epithelial cells and an increase in the thickness of the epithelial cell membrane, which affects functionality and promote fibrosis. These findings support our results, indicating that 2-AA mediates cholesterol accumulation, which may contribute to the pathogenesis driven by *PA*.

Our results, for the first time, provide evidence that the *PA*-derived 2-AA potentially exacerbates lung fibrosis by modulating key genes involved in mucus production, inflammation, and extracellular matrix remodeling in both endothelial and epithelial cells, which are crucial in CF and IPF. Interestingly, in HPMEC cells, the CF gene marker *CFTR expression* was lower. In contrast, other CF and IPF markers, *MUC5B, MUC4, and MUC16* genes, were higher, indicating hyper-concentrated mucus, ineffective mucociliary clearance, and impairment of the endothelial membrane barrier (103, 104). Furthermore, 2-AA treatment influenced pro-inflammatory responses in opposing ways between HPMEC and NHBE cells, suggesting a cell-specific mechanism of action. Studies have reported the critical role of endothelial cells in CF-related pathology, particularly in mediating inflammation and vascular remodelling (103, 105–107). These findings suggest that 2-AA can be a potential biomarker or therapeutic target in chronic lung diseases.

The findings presented reveal the previously unreported impact of the MvfR-regulated secreted molecule, 2-AA, on primary female bronchial airway epithelial cells and pulmonary endothelial cells. The resulting cellular changes may provide new insights into our understanding of lung *PA* infections in healthy individuals and those with CF and IPF. At the core of the findings in HPMEC and NHBE cells are the observed mitochondrial dysfunction, metabolic alterations, and distinct signaling pathways, providing crucial insights for developing cell-specific targeted countermeasures against *PA*.

## Data Availability

The data is publicly present with GEO accession: GSE290885 and following the link https://www.ncbi.nlm.nih.gov/geo/query/acc.cgi?acc=GSE290885.

## Supplementary material

Supplementary Figure 1 Principal component (PCA) analysis of untreated and 2-AA treated samples. **(A)** PCA plot of human pulmonary microvascular endothelial (HPMEC) cells (n=2). **(B)** PCA plot of normal human bronchial epithelial (NHBE) cells (n=2). A clear separation between untreated (PC1) and 2-AA treated (PC2) is observed. The genes were normalized using DeSeq2.

Supplementary Figure 2 2-AA suppresses pro-inflammatory cytokine secretion in HPMECs. Cytokine levels in cell culture supernatants of HPMEC treated with 2-AA for 20 hrs or PBS (control). **(A)** IL-6, **(B)** CCL-2, **(C)** CXCL-8, **(D)** TNF- α, **(E)** TGF-β, **(F)** IL-10 and **(G)** IL-4. Levels were normalized to total supernatant protein content (pg/mg protein). Data are presented as mean ± SD; each dot represents five different biological replicates (n = 5). An unpaired two-tailed t-test determined p-values. The overall p-value summary of the panel **(A-E)** is p < 0.0001, indicating significance, and that of the panel **(E, F)** is p < 0.45, indicating non-significance.

Supplementary Figure 3 Representative immunofluorescence microscopy of mice lung tissue sections 4 days post-2-AA treatment shows the accumulation of cholesterol. **(A)** Cholesterol accumulation was visualized using an antibody against the rate-limiting enzyme HMG-CoA reductase (HMGCR) (red). Nuclei (blue) were counterstained with DAPI. PBS-treated cells were used as a control. The scale bar represents 100 µm. **(B)** Representative images of Haematoxylin and Eosin (H&E) staining of mouse lung sections. **(a)** untreated mice depict normal lung histology. **(c)** 2-AA-treated mice show thickening of the epithelial cell membrane. The black boxes highlight **(b)** normal bronchial epithelial cells in untreated mice and **(d)** hypertrophy in the bronchial epithelial cells of 2-AA treated mice. Images represent n = 4 mice per group, with 6 tissue sections analyzed per mouse.

Supplementary Figure 4 Baseline gene expression of cystic fibrosis biomarkers in NHBE and HPMEC cells exposed to PBS (control). Table showing the differential expression of genes in NHBE and HPMEC cells, where the expression increased (blue) or decreased (red) by at least a 1.4-fold change with a p-value < 0.05. n=2, *p<0.05, **p<0.01, ***p<0.001. The cloud-computing Galaxy server (usegalaxy.org) was used for data analysis.

Supplementary Figure 5 Effect of 2-AA on HPMEC viability. Endothelial cells were treated with increasing concentrations of 2-AA (0, 20, 50, 100, and 200 μM) for 20 hrs. Cell viability was assessed using the MTT assay and expressed as a percentage relative to PBS-treated controls (0 μM, set at 100%). Data are presented as mean ± SD (n = 5 per group). No statistically significant differences in cell viability were observed across treatment groups (p > 0.05, unpaired two-tailed t-test; exact p-values indicated above bars).

Supplementary Table 1 Reactome pathway enrichment analysis of HPMEC and NHBE cells exposed to 2-AA. Table showing the upregulated and downregulated pathways with the associated genes in HPMEC and NHBE cells with p<0.05 and a False Discovery Rate (FDR<0.05). The count represents the number of genes associated with a pathway.

Supplementary Table 2 Non-common 2-AA regulated genes and associated pathways in HPMEC and NHBE cells. Table showing Reactome pathway enrichment analysis of upregulated genes (blue) and downregulated genes (red) with their associated pathways after 20 hrs of continuous flow of 20 µM 2-AA treatment in human microvascular endothelial (HPMEC) **(B)** and NHBE **(A)** cells. Significance is denoted by p<0.05 and FDR<0.05. The Reactome pathway enrichment analysis was done using the DAVID web server.

## References

[1] Aggarwal S Singh V Chakraborty A Cha S Dimitriou A de Crescenzo C. Skeletal muscle mitochondrial dysfunction mediated by Pseudomonas aeruginosa quorum-sensing transcription factor MvfR: reversing effects with anti-MvfR and mitochondrial-targeted compounds. Mbio. (2024) 15:e01292–24. doi: 10.1128/mbio.01292-24, PMID: 38860823 PMC11253625

[2] Arijit C Arunava B Singh VK Filip K Sujin C Oldham WM. The bacterial quorum sensing signal 2’-aminoacetophenone rewires immune cell bioenergetics through the Ppargc1a/Esrra axis to mediate tolerance to infection. eLife. (2024) 13. doi: 10.7554/eLife.97568.3, PMID: 39269443 PMC11398867

[3] Chakraborty A Kabashi A Wilk S Rahme LG. Quorum-sensing signaling molecule 2-aminoacetophenone mediates the persistence of pseudomonas aeruginosa in macrophages by interference with autophagy through epigenetic regulation of lipid biosynthesis. MBio. (2023) 14:e00159–23. doi: 10.1128/mbio.00159-23, PMID: 37010415 PMC10127747

[4] Deep A Chaudhary U Gupta V. Quorum sensing and bacterial pathogenicity: from molecules to disease. J Lab Physicians. (2011) 3:004–11. doi: 10.4103/0974-2727.78553, PMID: 21701655 PMC3118056

[5] Shiner E Terentyev D Bryan A Sennoune S Martinez-Zaguilan R Li G. Pseudomonas aeruginosa autoinducer modulates host cell responses through calcium signalling. Cell Microbiol. (2006) 8:1601–10. doi: 10.1111/j.1462-5822.2006.00734.x, PMID: 16984415

[6] Singh VK Almpani M Maura D Kitao T Ferrari L Fontana S. Tackling recalcitrant Pseudomonas aeruginosa infections in critical illness via anti-virulence monotherapy. Nat Commun. (2022) 13:5103. doi: 10.1038/s41467-022-32833-9, PMID: 36042245 PMC9428149

[7] Elfadadny A Ragab R AlHarbi M Badshah F Ibáñez-Arancibia E Farag A. Antimicrobial resistance of Pseudomonas aeruginosa: navigating clinical impacts, current resistance trends, and innovations in breaking therapies. Front Microbiol. (2024) 15:1–20. doi: 10.3389/fmicb.2024.1374466, PMID: 38646632 PMC11026690

[8] Qin S Xiao W Zhou C Pu Q Deng X Lan L. Pseudomonas aeruginosa: pathogenesis, virulence factors, antibiotic resistance, interaction with host, technology advances and emerging therapeutics. Signal Transduct Target Ther. (2022) 7:199. doi: 10.1038/s41392-022-01056-1, PMID: 35752612 PMC9233671

[9] Venkateswaran P Vasudevan S David H Shaktivel A Shanmugam K Neelakantan P. Revisiting ESKAPE Pathogens: Virulence, resistance, and combating strategies focusing on quorum sensing. Front Cell Infect Microbiol. (2023) 13:1159798. doi: 10.3389/fcimb.2023.1159798, PMID: 37457962 PMC10339816

[10] De Oliveira DM Forde BM Kidd TJ Harris PN Schembri MA Beatson SA. Antimicrobial resistance in ESKAPE pathogens. Clin Microbiol Rev. (2020) 33:10–1128. doi: 10.1128/cmr.00181-19, PMID: 32404435 PMC7227449

[11] Curran CS Bolig T Torabi-Parizi P. Mechanisms and targeted therapies for Pseudomonas aeruginosa lung infection. Am J Respir Crit Care Med. (2018) 197:708–27. doi: 10.1164/rccm.201705-1043SO, PMID: 29087211 PMC5855068

[12] Nickerson R Thornton CS Johnston B Lee AH Cheng Z. Pseudomonas aeruginosa in chronic lung disease: untangling the dysregulated host immune response. Front Immunol. (2024) 15:1405376. doi: 10.3389/fimmu.2024.1405376, PMID: 39015565 PMC11250099

[13] Renema P Hardy KS Housley N Dunbar G Annamdevula N Britain A. cAMP signaling primes lung endothelial cells to activate caspase-1 during Pseudomonas aeruginosa infection. Am J Physiol Lung Cell Mol Physiol. (2020) 318:L1074–L83. doi: 10.1152/ajplung.00185.2019, PMID: 32186399 PMC7272745

[14] Evans S Turner S Bosch B Hardy C Woodhead M. Lung function in bronchiectasis: the influence of Pseudomonas aeruginosa. Eur Respir J. (1996) 9:1601–4. doi: 10.1183/09031936.96.09081601, PMID: 8866579

[15] Koch C. Early infection and progression of cystic fibrosis lung disease. Pediatr Pulmonol. (2002) 34:232–6. doi: 10.1002/ppul.10135, PMID: 12203855

[16] Monso E Garcia-Aymerich J Soler N Farrero E Felez M Anto J. Bacterial infection in exacerbated COPD with changes in sputum characteristics. Epidemiol Infect. (2003) 131:799–804. doi: 10.1017/S0950268803008872, PMID: 12948381 PMC2870022

[17] Patel I Seemungal T Wilks M Lloyd-Owen S Donaldson G Wedzicha J. Relationship between bacterial colonisation and the frequency, character, and severity of COPD exacerbations. Thorax. (2002) 57:759–64. doi: 10.1136/thorax.57.9.759, PMID: 12200518 PMC1746426

[18] Nadal Jimenez P Koch G Thompson JA Xavier KB Cool RH Quax WJ. The multiple signaling systems regulating virulence in Pseudomonas aeruginosa. Microbiol Mol Biol Rev. (2012) 76:46–65. doi: 10.1128/MMBR.05007-11, PMID: 22390972 PMC3294424

[19] Bandyopadhaya A Kesarwani M Que Y-A He J Padfield K Tompkins R. The quorum sensing volatile molecule 2-amino acetophenon modulates host immune responses in a manner that promotes life with unwanted guests. PloS Pathogens. (2012) 8:e1003024. doi: 10.1371/journal.ppat.1003024, PMID: 23166496 PMC3499575

[20] Bandyopadhaya A Tsurumi A Maura D Jeffrey KL Rahme LG. A quorum-sensing signal promotes host tolerance training through HDAC1-mediated epigenetic reprogramming. Nat Microbiol. (2016) 1:1–9. doi: 10.1038/nmicrobiol.2016.174, PMID: 27694949 PMC5066596

[21] Bandyopadhaya A Tsurumi A Rahme LG. NF-κBp50 and HDAC1 interaction is implicated in the host tolerance to infection mediated by the bacterial quorum sensing signal 2-aminoacetophenone. Front Microbiol. (2017) 8:1211. doi: 10.3389/fmicb.2017.01211, PMID: 28713342 PMC5492500

[22] Cao H Krishnan G Goumnerov B Tsongalis J Tompkins R Rahme LG. A quorum sensing-associated virulence gene of Pseudomonas aeruginosa encodes a LysR-like transcription regulator with a unique self-regulatory mechanism. Proc Natl Acad Sci. (2001) 98:14613–8. doi: 10.1073/pnas.251465298, PMID: 11724939 PMC64730

[23] Déziel E Lépine F Milot S He J Mindrinos MN Tompkins RG. Analysis of Pseudomonas aeruginosa 4-hydroxy-2-alkylquinolines (HAQs) reveals a role for 4-hydroxy-2-heptylquinoline in cell-to-cell communication. Proc Natl Acad Sci. (2004) 101:1339–44. doi: 10.1073/pnas.0307694100, PMID: 14739337 PMC337054

[24] Kesarwani M Hazan R He J Que Y Apidianakis Y Lesic B. A quorum sensing regulated small volatile molecule reduces acute virulence and promotes chronic infection phenotypes. PloS Pathogens. (2011) 7:e1002192. doi: 10.1371/journal.ppat.1002192, PMID: 21829370 PMC3150319

[25] Que Y-A Hazan R Strobel B Maura D He J Kesarwani M. A quorum sensing small volatile molecule promotes antibiotic tolerance in bacteria. PloS One. (2013) 8:e80140. doi: 10.1371/journal.pone.0080140, PMID: 24367477 PMC3868577

[26] Hazan R Que YA Maura D Strobel B Majcherczyk PA Hopper LR. Auto poisoning of the respiratory chain by a quorum-sensing-regulated molecule favors biofilm formation and antibiotic tolerance. Curr Biol. (2016) 26:195–206. doi: 10.1016/j.cub.2015.11.056, PMID: 26776731 PMC4729643

[27] Scott-Thomas AJ Syhre M Pattemore PK Epton M Laing R Pearson J. 2-Aminoacetophenone as a potential breath biomarker for Pseudomonas aeruginosa in the cystic fibrosis lung. BMC Pulmon Med. (2010) 10:1–10. doi: 10.1186/1471-2466-10-56, PMID: 21054900 PMC2989937

[28] Bishai JD Palm NW. Small molecule metabolites at the host–microbiota interface. J Immunol. (2021) 207:1725–33. doi: 10.4049/jimmunol.2100528, PMID: 34544815 PMC8500551

[29] Donia MS Fischbach MA. Small molecules from the human microbiota. Science. (2015) 349:1254766. doi: 10.1126/science.1254766, PMID: 26206939 PMC4641445

[30] Komarova YA Kruse K Mehta D Malik AB. Protein interactions at endothelial junctions and signaling mechanisms regulating endothelial permeability. Circ Res. (2017) 120:179–206. doi: 10.1161/CIRCRESAHA.116.306534, PMID: 28057793 PMC5225667

[31] Siggins MK Sriskandan S. Bacterial lymphatic metastasis in infection and immunity. Cells. (2022) 11:33. doi: 10.3390/cells11010033, PMID: 35011595 PMC8750085

[32] Bandyopadhaya A Constantinou C Psychogios N Ueki R Yasuhara S Martyn JJ. Bacterial-excreted small volatile molecule 2-aminoacetophenone induces oxidative stress and apoptosis in murine skeletal muscle. Int J Mol Med. (2016) 37:867–78. doi: 10.3892/ijmm.2016.2487, PMID: 26935176 PMC4790710

[33] Bandyopadhaya A Tzika AA Rahme LG. Pseudomonas aeruginosa quorum sensing molecule alters skeletal muscle protein homeostasis by perturbing the antioxidant defense system. MBio. (2019) 10:10–1128. doi: 10.1128/mbio.02211-19, PMID: 31575771 PMC6775459

[34] Tzika AA Constantinou C Bandyopadhaya A Psychogios N Lee S Mindrinos M. A small volatile bacterial molecule triggers mitochondrial dysfunction in murine skeletal muscle. PloS One. (2013) 8:e74528. doi: 10.1371/journal.pone.0074528, PMID: 24098655 PMC3787027

[35] Baptista LS Porrini C Kronemberger GS Kelly DJ Perrault CM. 3D organ-on-a-chip: The convergence of microphysiological systems and organoids. Front Cell Dev Biol. (2022) 10:1043117. doi: 10.3389/fcell.2022.1043117, PMID: 36478741 PMC9720174

[36] Ko J Park D Lee S Gumuscu B Jeon NL. Engineering organ-on-a-chip to accelerate translational research. Micromachines. (2022) 13:1200. doi: 10.3390/mi13081200, PMID: 36014122 PMC9412404

[37] Nithin R Aggarwal A Sravani AB Mallya P Lewis S. Organ-on-a-chip: an emerging research platform. Organogenesis. (2023) 19:2278236. doi: 10.1080/15476278.2023.2278236, PMID: 37965897 PMC10653779

[38] Chou DB Frismantas V Milton Y David R Pop-Damkov P Ferguson D. On-chip recapitulation of clinical bone marrow toxicities and patient-specific pathophysiology. Nat Biomed Eng. (2020) 4:394–406. doi: 10.1038/s41551-019-0495-z, PMID: 31988457 PMC7160021

[39] Herland A Maoz BM Das D Somayaji MR Prantil-Baun R Novak R. Quantitative prediction of human pharmacokinetic responses to drugs via fluidically coupled vascularized organ chips. Nat Biomed Eng. (2020) 4:421–36. doi: 10.1038/s41551-019-0498-9, PMID: 31988459 PMC8011576

[40] Huh D Leslie DC Matthews BD Fraser JP Jurek S Hamilton GA. A human disease model of drug toxicity–induced pulmonary edema in a lung-on-a-chip microdevice. Sci Trans Med. (2012) 4:159ra47–ra47. doi: 10.1126/scitranslmed.3004249, PMID: 23136042 PMC8265389

[41] Huh D Matthews BD Mammoto A Montoya-Zavala M Hsin HY Ingber DE. Reconstituting organ-level lung functions on a chip. Science. (2010) 328:1662–8. doi: 10.1126/science.1188302, PMID: 20576885 PMC8335790

[42] Prantil-Baun R Novak R Das D Somayaji MR Przekwas A Ingber DE. Physiologically based pharmacokinetic and pharmacodynamic analysis enabled by microfluidically linked organs-on-chips. Annu Rev Pharmacol Toxicol. (2018) 58:37–64. doi: 10.1146/annurev-pharmtox-010716-104748, PMID: 29309256

[43] Ahmed DW Eiken MK DePalma SJ Helms AS Zemans RL Spence JR. Integrating mechanical cues with engineered platforms to explore cardiopulmonary development and disease. IScience. (2023) 26:108472. doi: 10.1016/j.isci.2023.108472, PMID: 38077130 PMC10698280

[44] Bennet TJ Randhawa A Hua J Cheung KC. Airway-on-a-chip: designs and applications for lung repair and disease. Cells. (2021) 10:1602. doi: 10.3390/cells10071602, PMID: 34206722 PMC8304815

[45] Leung CM De Haan P Ronaldson-Bouchard K Kim G-A Ko J Rho HS. A guide to the organ-on-a-chip. Nat Rev Methods Primers. (2022) 2:33. doi: 10.1038/s43586-022-00118-6

[46] Vasil ML Stonehouse MJ Vasil AI Wadsworth SJ Goldfine H Bolcome RE III. A complex extracellular sphingomyelinase of Pseudomonas aeruginosa inhibits angiogenesis by selective cytotoxicity to endothelial cells. PloS Pathogens. (2009) 5:e1000420. doi: 10.1371/journal.ppat.1000420, PMID: 19424430 PMC2673038

[47] Afgan E Baker D Van den Beek M Blankenberg D Bouvier D Čech M. The Galaxy platform for accessible, reproducible and collaborative biomedical analyses: 2016 update. Nucleic Acids Res. (2016) 44:W3–W10. doi: 10.1093/nar/gkw343, PMID: 27137889 PMC4987906

[48] Jovanovic DV Di Battista JA Martel-Pelletier J Jolicoeur FC He Y Zhang M. IL-17 stimulates the production and expression of proinflammatory cytokines, IL-β and TNF-α, by human macrophages. J Immunol. (1998) 160:3513–21. doi: 10.4049/jimmunol.160.7.3513, PMID: 9531313

[49] Lacy P. Mechanisms of degranulation in neutrophils. Allergy Asthma Clin Immunol. (2006) 2:1–11. doi: 10.1186/1710-1492-2-3-98, PMID: 20525154 PMC2876182

[50] Lu P Takai K Weaver VM Werb Z. Extracellular matrix degradation and remodeling in development and disease. Cold Spring Harbor Perspect Biol. (2011) 3:a005058. doi: 10.1101/cshperspect.a005058, PMID: 21917992 PMC3225943

[51] Vermeer PD Denker J Estin M Moninger TO Keshavjee S Karp P. MMP9 modulates tight junction integrity and cell viability in human airway epithelia. Am J Physiol Lung Cell Mol Physiol. (2009) 296:L751–L62. doi: 10.1152/ajplung.90578.2008, PMID: 19270179 PMC2681350

[52] Florence JM Krupa A Booshehri LM Allen TC Kurdowska AK. Metalloproteinase-9 contributes to endothelial dysfunction in atherosclerosis via protease activated receptor-1. PloS One. (2017) 12:e0171427. doi: 10.1371/journal.pone.0171427, PMID: 28166283 PMC5293219

[53] Pickett JR Wu Y Zacchi LF Ta HT. Targeting endothelial vascular cell adhesion molecule-1 in atherosclerosis: drug discovery and development of vascular cell adhesion molecule-1–directed novel therapeutics. Cardiovasc Res. (2023) 119:2278–93. doi: 10.1093/cvr/cvad130, PMID: 37595265 PMC10597632

[54] Heinosalo T Saarinen N Poutanen M. Role of hydroxysteroid (17beta) dehydrogenase type 1 in reproductive tissues and hormone-dependent diseases. Mol Cell Endocrinol. (2019) 489:9–31. doi: 10.1016/j.mce.2018.08.004, PMID: 30149044

[55] Castillo-Sánchez JC Cruz A Pérez-Gil J. Structural hallmarks of lung surfactant: Lipid-protein interactions, membrane structure and future challenges. Arch Biochem Biophys. (2021) 703:108850. doi: 10.1016/j.abb.2021.108850, PMID: 33753033

[56] de la Serna JB Perez-Gil J Simonsen AC Bagatolli LA. Cholesterol rules: direct observation of the coexistence of two fluid phases in native pulmonary surfactant membranes at physiological temperatures. J Biol Chem. (2004) 279:40715–22. doi: 10.1074/jbc.M404648200, PMID: 15231828

[57] Maddocks SE Oyston PC. Structure and function of the LysR-type transcriptional regulator (LTTR) family proteins. Microbiology. (2008) 154:3609–23. doi: 10.1099/mic.0.2008/022772-0, PMID: 19047729

[58] Evans CM Fingerlin TE Schwarz MI Lynch D Kurche J Warg L. Idiopathic pulmonary fibrosis: a genetic disease that involves mucociliary dysfunction of the peripheral airways. Physiol Rev. (2016) 96:1567–91. doi: 10.1152/physrev.00004.2016, PMID: 27630174 PMC5243224

[59] Earnest A Salimi F Wainwright CE Bell SC Ruseckaite R Ranger T. Lung function over the life course of paediatric and adult patients with cystic fibrosis from a large multi-centre registry. Sci Rep. (2020) 10:17421. doi: 10.1038/s41598-020-74502-1, PMID: 33060788 PMC7567842

[60] Sousa L Pankonien I Clarke LA Silva I Kunzelmann K Amaral MD. KLF4 Acts as a wt-CFTR Suppressor through an AKT-Mediated Pathway. Cells. (2020) 9:1607. doi: 10.3390/cells9071607, PMID: 32630830 PMC7408019

[61] Kroncke BM Van Horn WD Smith J Kang C Welch RC Song Y. Structural basis for KCNE3 modulation of potassium recycling in epithelia. Sci Adv. (2016) 2:e1501228. doi: 10.1126/sciadv.1501228, PMID: 27626070 PMC5017827

[62] Crambert G. HK-ATPase type 2: relevance for renal physiology and beyond. Am J Physiol Renal Physiol. (2014) 306:F693–700. doi: 10.1152/ajprenal.00605.2013, PMID: 24431203

[63] Caslin HL Abebayehu D Pinette JA Ryan JJ. Lactate is a metabolic mediator that shapes immune cell fate and function. Front Physiol. (2021) 12:688485. doi: 10.3389/fphys.2021.688485, PMID: 34733170 PMC8558259

[64] Bodas M Vij N. The NFκB signaling in cystic fibrosis lung disease: pathophysiology and therapeutic potential. Discov Med. (2010) 9:346., PMID: 20423679 PMC3114405

[65] Bonfield TL Panuska JR Konstan MW Hilliard KA Hilliard JB Ghnaim H. Inflammatory cytokines in cystic fibrosis lungs. Am J Respir Crit Care Med. (1995) 152:2111–8. doi: 10.1164/ajrccm.152.6.8520783, PMID: 8520783

[66] Courtney J Ennis M Elborn J. Cytokines and inflammatory mediators in cystic fibrosis. J Cystic Fibrosis. (2004) 3:223–31. doi: 10.1016/j.jcf.2004.06.006, PMID: 15698939

[67] Martin A Laing I Zhang G Brennan S Winfield K Sly P. CD14 C-159T and early infection with Pseudomonas aeruginosa in children with cystic fibrosis. Respir Res. (2005) 6:1–4. doi: 10.1186/1465-9921-6-63, PMID: 15975149 PMC1168907

[68] Scagnolari C Bitossi C Frasca F Viscido A Brazzini G Trancassini M. Differential toll like receptor expression in cystic fibrosis patients’ airways during rhinovirus infection. J Infect. (2020) 81:726–35. doi: 10.1016/j.jinf.2020.07.021, PMID: 32712204

[69] Foell D Seeliger S Vogl T Koch H Maschek H Harms E. Expression of S100A12 (EN-RAGE) in cystic fibrosis. Thorax. (2003) 58:613–7. doi: 10.1136/thorax.58.7.613, PMID: 12832680 PMC1746749

[70] Hunt WR Helfman BR McCarty NA Hansen JM. Advanced glycation end products are elevated in cystic fibrosis-related diabetes and correlate with worse lung function. J Cystic Fibrosis. (2016) 15:681–8. doi: 10.1016/j.jcf.2015.12.011, PMID: 26817932

[71] Tirkos S Newbigging S Nguyen V Keet M Ackerley C Kent G. Expression of S100A8 correlates with inflammatory lung disease in congenic mice deficient of the cystic fibrosis transmembrane conductance regulator. Respir Res. (2006) 7:1–11. doi: 10.1186/1465-9921-7-51, PMID: 16571124 PMC1456967

[72] Idris T Bachmann M Bacchetta M Bernhard W-H Chanson M Badaoui M. Akt-driven TGF-β and DKK1 secretion impairs F508del cystic fibrosis airway epithelium polarity. Am J Respir Cell Mol Biol. (2024) 71:81–94. doi: 10.1165/rcmb.2023-0408OC, PMID: 38531016

[73] Kramer EL Clancy JP. TGFβ as a therapeutic target in cystic fibrosis. Expert Opin Ther Targets. (2018) 22:177–89. doi: 10.1080/14728222.2018.1406922, PMID: 29168406 PMC6094931

[74] Gu Y Harley IT Henderson LB Aronow BJ Vietor I Huber LA. Identification of IFRD1 as a modifier gene for cystic fibrosis lung disease. Nature. (2009) 458:1039–42. doi: 10.1038/nature07811, PMID: 19242412 PMC2841516

[75] Gaggar A Hector A Bratcher PE Mall MA Griese M Hartl D. The role of matrix metalloproteases in cystic fibrosis lung disease. Eur Respir J. (2011) 38:721–7. doi: 10.1183/09031936.00173210, PMID: 21233269 PMC4036453

[76] Nagase H Woessner JF Jr. Matrix metalloproteinases *. J Biol Chem. (1999) 274:21491–4. doi: 10.1074/jbc.274.31.21491, PMID: 10419448

[77] Sonnenberg-Riethmacher E Miehe M Riethmacher D. Periostin in allergy and inflammation. Front Immunol. (2021) 12:722170. doi: 10.3389/fimmu.2021.722170, PMID: 34512647 PMC8429843

[78] Rojas-Quintero J Owen CA. Matrix metalloproteinases in cystic fibrosis: pathophysiologic and therapeutic perspectives. Metalloproteinases In Med. (2016) 3:49–62.

[79] Schupp JC Khanal S Gomez JL Sauler M Adams TS Chupp GL. Single-cell transcriptional archetypes of airway inflammation in cystic fibrosis. Am J Respir Crit Care Med. (2020) 202:1419–29. doi: 10.1164/rccm.202004-0991OC, PMID: 32603604 PMC7667912

[80] Kormann MS Dewerth A Eichner F Baskaran P Hector A Regamey N. Transcriptomic profile of cystic fibrosis patients identifies type I interferon response and ribosomal stalk proteins as potential modifiers of disease severity. PloS One. (2017) 12:e0183526. doi: 10.1371/journal.pone.0183526, PMID: 28846703 PMC5573219

[81] Gokey JJ Snowball J Sridharan A Speth JP Black KE Hariri LP. MEG3 is increased in idiopathic pulmonary fibrosis and regulates epithelial cell differentiation. JCI Insight. (2018) 3:e122490. doi: 10.1172/jci.insight.122490, PMID: 30185671 PMC6171798

[82] Al Hamwi G Namasivayam V Büschbell B Gedschold R Golz S Müller CE. Proinflammatory chemokine CXCL14 activates MAS-related G protein-coupled receptor MRGPRX2 and its putative mouse ortholog MRGPRB2. Commun Biol. (2024) 7:52. doi: 10.1038/s42003-023-05739-5, PMID: 38184723 PMC10771525

[83] Stainer A Faverio P Busnelli S Catalano M Della Zoppa M Marruchella A. Molecular biomarkers in idiopathic pulmonary fibrosis: state of the art and future directions. Int J Mol Sci. (2021) 22:6255. doi: 10.3390/ijms22126255, PMID: 34200784 PMC8230407

[84] Renaud L Da Silveira WA Takamura N Hardiman G Feghali-Bostwick C. Prominence of IL6, IGF, TLR, and bioenergetics pathway perturbation in lung tissues of scleroderma patients with pulmonary fibrosis. Front Immunol. (2020) 11:383. doi: 10.3389/fimmu.2020.00383, PMID: 32210969 PMC7075854

[85] Jiang P Gil de Rubio R Hrycaj SM Gurczynski SJ Riemondy KA Moore BB. Ineffectual type 2–to–type 1 alveolar epithelial cell differentiation in idiopathic pulmonary fibrosis: persistence of the KRT8hi transitional state. Am J Respir Crit Care Med. (2020) 201:1443–7. doi: 10.1164/rccm.201909-1726LE, PMID: 32073903 PMC7258651

[86] Ballester B Milara J Montero P Cortijo J. MUC16 is overexpressed in idiopathic pulmonary fibrosis and induces fibrotic responses mediated by transforming growth factor-β1 canonical pathway. Int J Mol Sci. (2021) 22:6502. doi: 10.3390/ijms22126502, PMID: 34204432 PMC8235375

[87] Wang L Zhu M Li Y Yan P Li Z Chen X. Serum proteomics identifies biomarkers associated with the pathogenesis of idiopathic pulmonary fibrosis. Mol Cell Proteomics. (2023) 22:100524. doi: 10.1016/j.mcpro.2023.100524, PMID: 36870568 PMC10113895

[88] Barnett SN Cujba A-M Yang L Maceiras AR Li S Kedlian V. An organotypic atlas of human vascular cells. Nat Med. (2024) 30:1–. doi: 10.1038/s41591-024-03376-x, PMID: 39566559 PMC11645277

[89] Song L Li K Chen H Xie L. Cell cross-talk in alveolar microenvironment: from lung injury to fibrosis. Am J Respir Cell Mol Biol. (2024) 71:30–42. doi: 10.1165/rcmb.2023-0426TR, PMID: 38579159 PMC11225874

[90] Krüger-Genge A Blocki A Franke R-P Jung F. Vascular endothelial cell biology: an update. Int J Mol Sci. (2019) 20:4411. doi: 10.3390/ijms20184411, PMID: 31500313 PMC6769656

[91] Montgomery A Tam F Gursche C Cheneval C Besler K Enns W. Overlapping and distinct biological effects of IL-6 classic and trans-signaling in vascular endothelial cells. Am J Physiol Cell Physiol. (2021) 320:C554–C65. doi: 10.1152/ajpcell.00323.2020, PMID: 33471622

[92] Sawant DA Tharakan B Wilson RL Stagg HW Hunter FA Childs EW. Regulation of tumor necrosis factor-α–induced microvascular endothelial cell hyperpermeability by recombinant B-cell lymphoma-extra large. J Surg Res. (2013) 184:628–37. doi: 10.1016/j.jss.2013.04.079, PMID: 23731686 PMC3759616

[93] Tanaka T Narazaki M Kishimoto T. IL-6 in inflammation, immunity, and disease. Cold Spring Harbor Perspect Biol. (2014) 6:a016295. doi: 10.1101/cshperspect.a016295, PMID: 25190079 PMC4176007

[94] Bochenek ML Saar K Nazari-Jahantigh M Gogiraju R Wiedenroth CB Münzel T. Endothelial overexpression of TGF-β-induced protein impairs venous thrombus resolution: possible role in CTEPH. Basic to Trans Sci. (2024) 9:100–16. doi: 10.1038/s41591-024-03376-x, PMID: 38362348 PMC10864968

[95] Sage H Trüeb B Bornstein P. Biosynthetic and structural properties of endothelial cell type VIII collagen. J Biol Chem. (1983) 258:13391–401. doi: 10.1016/S0021-9258(17)44129-9 6630235

[96] Wang H Su Y. Collagen IV contributes to nitric oxide-induced angiogenesis of lung endothelial cells. Am J Physiol Cell Physiol. (2011) 300:C979–C88. doi: 10.1152/ajpcell.00368.2010, PMID: 21307347 PMC3093940

[97] Sharma A Ahmad S Ahmad T Ali S Syed MA. Mitochondrial dynamics and mitophagy in lung disorders. Life Sci. (2021) 284:119876. doi: 10.1016/j.lfs.2021.119876, PMID: 34389405

[98] He J Xiu F Chen Y Yang Y Liu H Xi Y. Aerobic glycolysis of bronchial epithelial cells rewires Mycoplasma pneumoniae pneumonia and promotes bacterial elimination. Infect Immun. (2024) 92:e00248–23. doi: 10.1128/iai.00248-23, PMID: 38205952 PMC10863416

[99] Keating E Rahman L Francis J Petersen A Possmayer F Veldhuizen R. Effect of cholesterol on the biophysical and physiological properties of a clinical pulmonary surfactant. Biophys J. (2007) 93:1391–401. doi: 10.1529/biophysj.106.099762, PMID: 17526587 PMC1929052

[100] Fessler MB Summer RS. Surfactant lipids at the host–environment interface. Metabolic sensors, suppressors, and effectors of inflammatory lung disease. Am J Respir Cell Mol Biol. (2016) 54:624–35. doi: 10.1165/rcmb.2016-0011PS, PMID: 26859434 PMC4942198

[101] Thacker VV Dhar N Sharma K Barrile R Karalis K McKinney JD. A lung-on-chip model of early Mycobacterium tuberculosis infection reveals an essential role for alveolar epithelial cells in controlling bacterial growth. Elife. (2020) 9:e59961. doi: 10.7554/eLife.59961, PMID: 33228849 PMC7735758

[102] Fessler MB. A new frontier in immunometabolism. Cholesterol in lung health and disease. Ann Am Thorac Soc. (2017) 14:S399–405. doi: 10.1513/AnnalsATS.201702-136AW, PMID: 29161079 PMC5711269

[103] Declercq M Treps L Carmeliet P Witters P. The role of endothelial cells in cystic fibrosis. J Cystic Fibrosis. (2019) 18:752–61. doi: 10.1016/j.jcf.2019.07.005, PMID: 31401006

[104] Matsui H Grubb BR Tarran R Randell SH Gatzy JT Davis CW. Evidence for periciliary liquid layer depletion, not abnormal ion composition, in the pathogenesis of cystic fibrosis airways disease. Cell. (1998) 95:1005–15. doi: 10.1016/S0092-8674(00)81724-9, PMID: 9875854

[105] Plebani R Tripaldi R Lanuti P Recchiuti A Patruno S Di Silvestre S. Establishment and long-term culture of human cystic fibrosis endothelial cells. Lab Invest. (2017) 97:1375–84. doi: 10.1038/labinvest.2017.74, PMID: 28759010

[106] Declercq M de Zeeuw P Conchinha NV Geldhof V Ramalho AS García-Caballero M. Transcriptomic analysis of CFTR-impaired endothelial cells reveals a pro-inflammatory phenotype. Eur Respir J. (2021) 57:2000261. doi: 10.1183/13993003.00261-2020, PMID: 33184117

[107] Totani L Plebani R Piccoli A Di Silvestre S Lanuti P Recchiuti A. Mechanisms of endothelial cell dysfunction in cystic fibrosis. Biochim Biophys Acta (BBA) Mol Basis Dis. (2017) 1863:3243–53. doi: 10.1016/j.bbadis.2017.08.011, PMID: 28847515

