# Supplementary figures and images for: Revealing the impact of Pseudomonas aeruginosa quorum sensing molecule 2’-aminoacetophenone on the human bronchial-airway epithelium and pulmonary endothelium using a human airway-on-a-chip
Source: Front Immunol. 2025 Jul 15;16:1592597. doi: 10.3389/fimmu.2025.1592597 (PMC12305196; doi:10.3389/fimmu.2025.1592597)

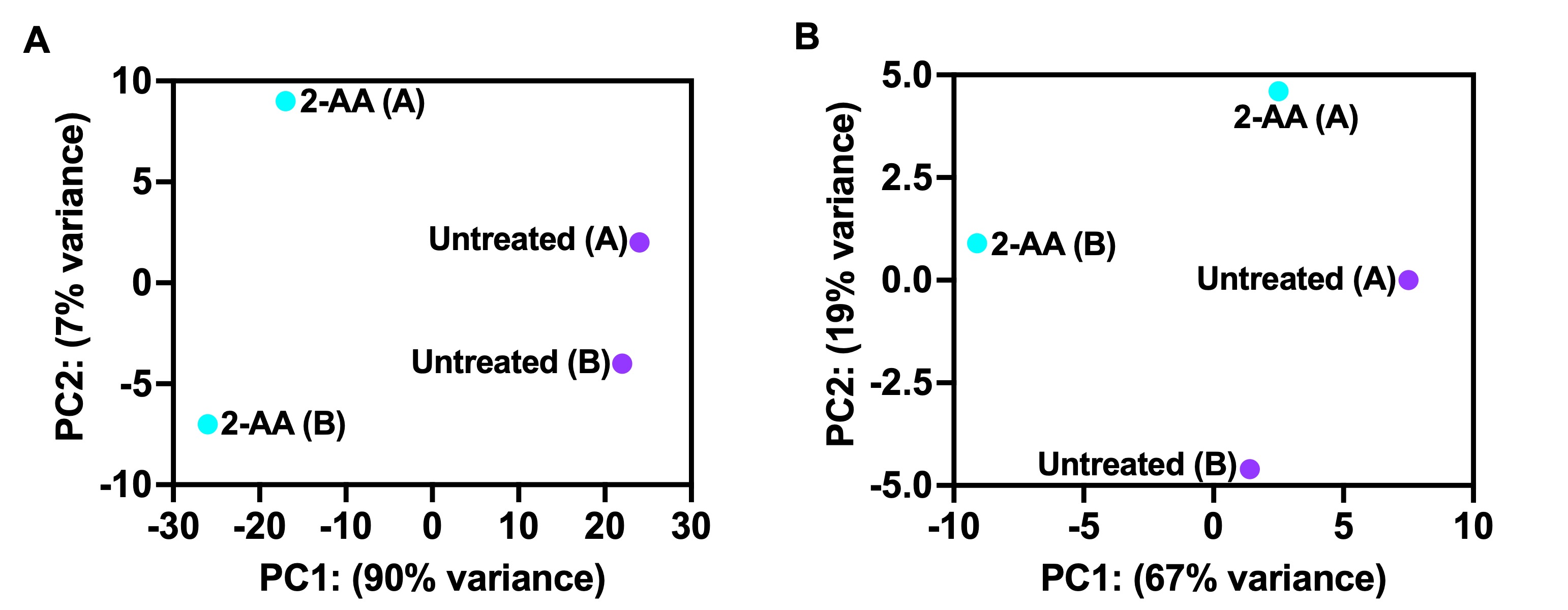

Supplement: Supplementary Figure 1 — Principal component (PCA) analysis of untreated and 2-AA treated samples. (A) PCA plot of human pulmonary microvascular endothelial (HPMEC) cells (n=2). (B) PCA plot of normal human bronchial epithelial (NHBE) cells (n=2). A clear separation between untreated (PC1) and 2-AA treated (PC2) is observed. The genes were normalized using DeSeq2. [file Image1.jpeg]

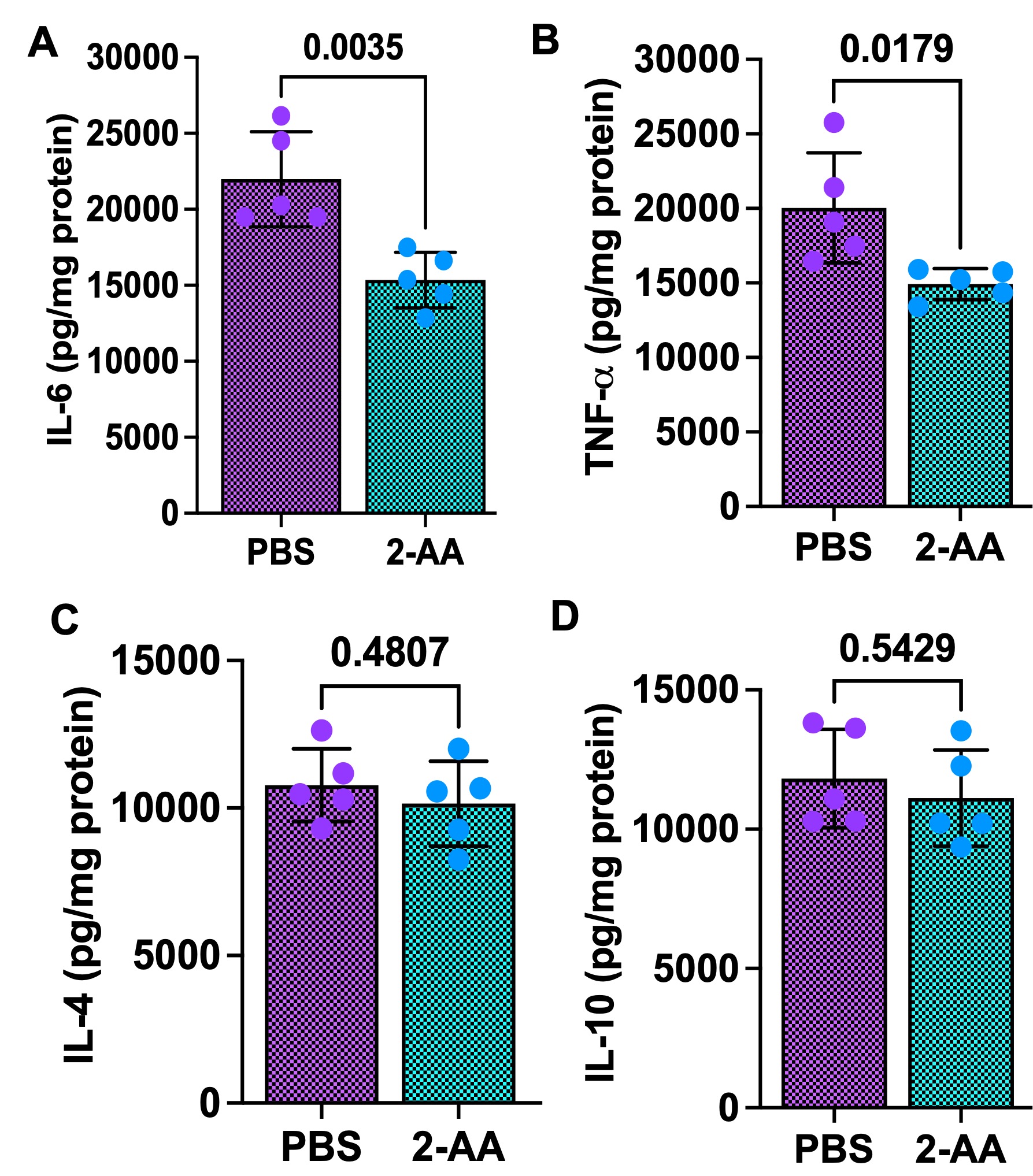

Supplement: Supplementary Figure 2 — 2-AA suppresses pro-inflammatory cytokine secretion in HPMECs. Cytokine levels in cell culture supernatants of HPMEC treated with 2-AA for 20 hrs or PBS (control). (A) IL-6, (B) CCL-2, (C) CXCL-8, (D) TNF- α, (E) TGF-β, (F) IL-10 and (G) IL-4. Levels were normalized to total supernatant protein content (pg/mg protein). Data are presented as mean ± SD; each dot represents five different biological replicates (n = 5). An unpaired two-tailed t-test determined p-values. The overall p-value summary of the panel (A-E) is p < 0.0001, indicating significance, and that of the panel (E, F) is p < 0.45, indicating non-significance. [file Image2.jpeg]

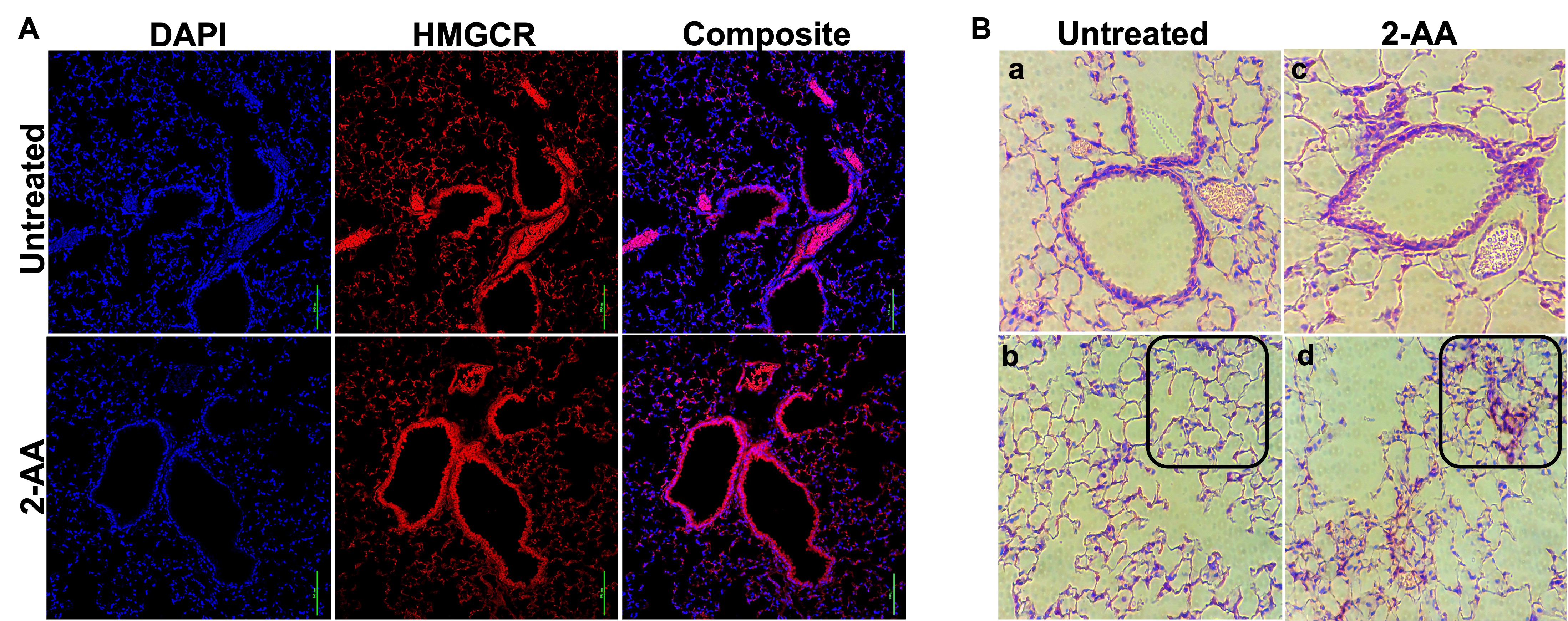

Supplement: Supplementary Figure 3 — Representative immunofluorescence microscopy of mice lung tissue sections 4 days post-2-AA treatment shows the accumulation of cholesterol. (A) Cholesterol accumulation was visualized using an antibody against the rate-limiting enzyme HMG-CoA reductase (HMGCR) (red). Nuclei (blue) were counterstained with DAPI. PBS-treated cells were used as a control. The scale bar represents 100 µm. (B) Representative images of Haematoxylin and Eosin (H&E) staining of mouse lung sections. (a) untreated mice depict normal lung histology. (c) 2-AA-treated mice show thickening of the epithelial cell membrane. The black boxes highlight (b) normal bronchial epithelial cells in untreated mice and (d) hypertrophy in the bronchial epithelial cells of 2-AA treated mice. Images represent n = 4 mice per group, with 6 tissue sections analyzed per mouse. [file Image3.jpeg]

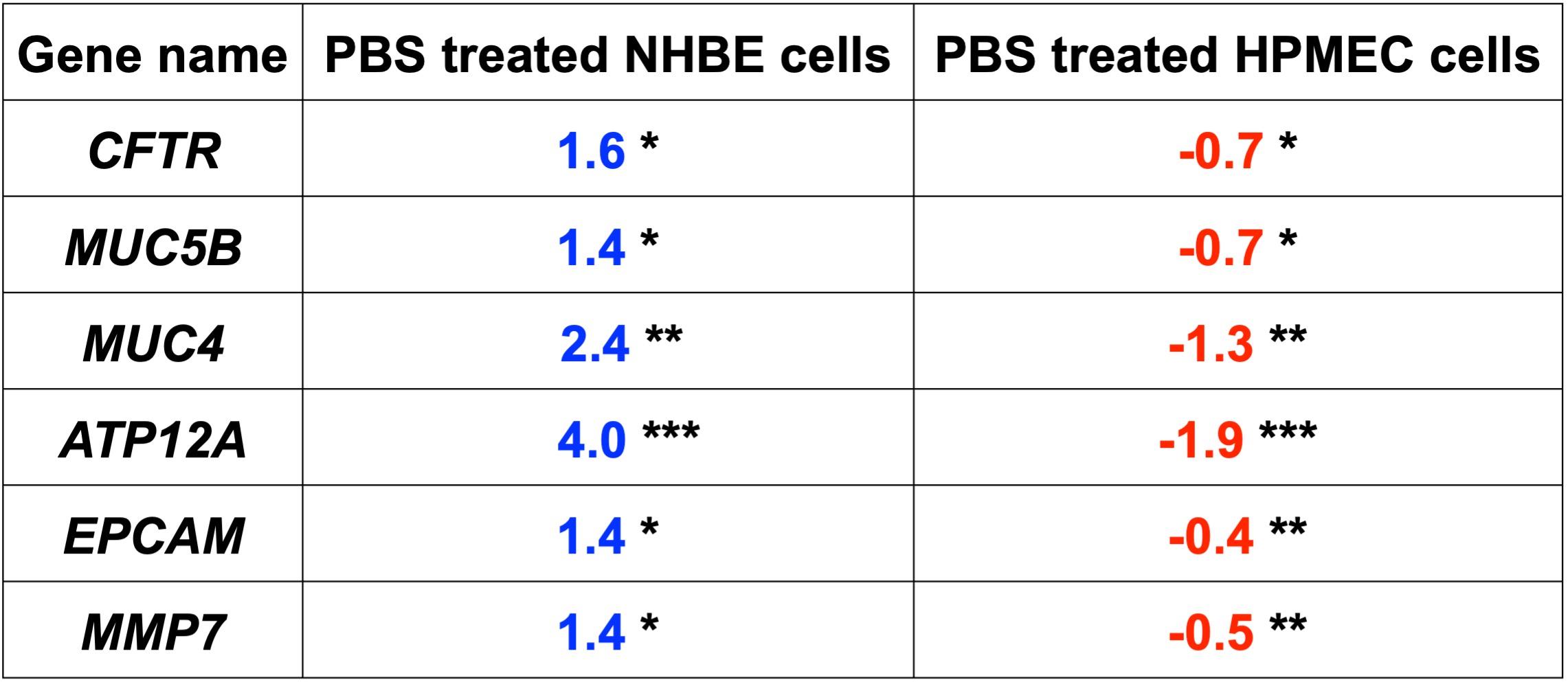

Supplement: Supplementary Figure 4 — Baseline gene expression of cystic fibrosis biomarkers in NHBE and HPMEC cells exposed to PBS (control). Table showing the differential expression of genes in NHBE and HPMEC cells, where the expression increased (blue) or decreased (red) by at least a 1.4-fold change with a p-value < 0.05. n=2, *p<0.05, **p<0.01, ***p<0.001. The cloud-computing Galaxy server (usegalaxy.org) was used for data analysis. [file Image4.jpeg]

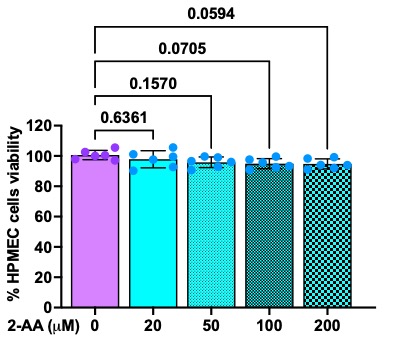

Supplement: Supplementary Figure 5 — Effect of 2-AA on HPMEC viability. Endothelial cells were treated with increasing concentrations of 2-AA (0, 20, 50, 100, and 200 μM) for 20 hrs. Cell viability was assessed using the MTT assay and expressed as a percentage relative to PBS-treated controls (0 μM, set at 100%). Data are presented as mean ± SD (n = 5 per group). No statistically significant differences in cell viability were observed across treatment groups (p > 0.05, unpaired two-tailed t-test; exact p-values indicated above bars). [file Image5.jpeg]
